# Electrodeposition: An efficient method to fabricate self‐supported electrodes for electrochemical energy conversion systems

**DOI:** 10.1002/EXP.20210077

**Published:** 2022-04-11

**Authors:** Junhyeong Kim, Hyunki Kim, Gyeong Ho Han, Seokjin Hong, Juhae Park, Junbeom Bang, Soo Young Kim, Sang Hyun Ahn

**Affiliations:** ^1^ School of Chemical Engineering and Material Science Chung‐Ang University Seoul Republic of Korea; ^2^ Department of Materials Science and Engineering Korea University Seoul Republic of Korea

**Keywords:** electrochemical energy conversion, electrodeposition, self‐supported electrode

## Abstract

The development of electrocatalysts for energy conversion systems is essential for alleviating environmental problems and producing useful energy sources as alternatives to fossil fuels. Improving the catalytic performance and stability of electrocatalysts is a major challenge in the development of energy conversion systems. Moreover, understanding their electrode structure is important for enhancing the energy efficiency. Recently, binder‐free self‐supported electrodes have been investigated because the seamless contact between the electrocatalyst and substrate minimizes the contact resistance as well as facilitates fast charge transfer at the catalyst/substrate interface and high catalyst utilization. Electrodeposition is an effective and facile method for fabricating self‐supported electrodes in aqueous solutions under mild conditions. Facile fabrication without a polymer binder and controlability of the compositional and morphological properties of the electrocatalyst make electrodeposition methods suitable for enhancing the performance of energy conversion systems. Herein, we summarize recent research on self‐supported electrodes fabricated by electrodeposition for energy conversion reactions, particularly focusing on cathodic reactions of electrolyzer system such as hydrogen evolution, electrochemical CO_2_ reduction, and electrochemical N_2_ reduction reactions. The deposition conditions, morphological and compositional properties, and catalytic performance of the electrocatalyst are reviewed. Finally, the prospective directions of electrocatalyst development for energy conversion systems are discussed.

## Graphical abstract

The high‐performance electrocatalysts have attracted significant attention to develop efficient energy conversion systems for clean production of hydrogen, hydrocarbon compounds, and ammonia. Electrodeposition is a simple method for fabricating the electrocatalysts with facile control of compositional and morphological properties in aqueous solutions under mild conditions. In this review, recent progress in electrodeposited electrocatalysts is summarized.

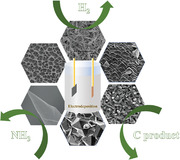

## 1 INTRODUCTION

Currently, 89% of the global energy demand is supplied by fossil fuels, and their indiscriminate use causes a gradual increase in the atmospheric CO_2_ concentration, accelerating global warming.^[^ ^1^, ^2^, ^3^, ^4^ ^]^ Moreover, the combustion of fossil fuels emits CO_2_ as well as air pollutants (e.g., NO*_x_* and SO*_x_*), which cause serious environmental problems.^[^ ^3^ ^]^ As a countermeasure, electrochemical energy conversion systems have been proposed.^[^ ^5^ ^]^ Using electricity, raw materials (e.g., water, carbon dioxide, and nitrogen) can be converted to value‐added fuels such as hydrogen, hydrocarbon compounds, and ammonia.^[^ ^6^, ^7^, ^8^, ^9^ ^]^ Compared with conventional methods, these systems have several advantages to achieve high efficiency of system operation (low operating temperature and pressure), high selectivity of desired product, and zero emission of greenhouse gas. Since the scale‐up of systems is feasible to obtain massive production of valuable fuels, several companies including ITM Power, General Electric, CETH2, and Siemens are exploring the possibility of commercialized energy conversion systems.^[^ ^10^, ^11^ ^]^ Moreover, when the systems are operated by electricity generated from renewable energies, entire process for fuel production becomes an eco‐friendly^[^ ^12^ ^]^ and the intermittent renewable energies can be stored as transportable fuels.^[^ ^13^ ^]^

High‐performance electrocatalysts are essential components for increasing the efficiency of energy conversion systems. For decades, extensive research has been conducted on strategies combining compositional and morphological controls to develop high‐performance electrocatalysts.^[^ ^14^ ^]^ It is known that the high intrinsic activity of an electrocatalyst can be achieved by the suitable binding energy of certain intermediates in the reaction mechanism.^[^ ^15^, ^16^, ^17^ ^]^ To this end, various approaches have been used to modulate the electronic structure via the fabrication of alloys, compounds, heterostructures, overlayers, core–shell structures, etc.^[^ ^18^, ^19^, ^20^ ^]^ Generating a number of active sites is also an important criterion for achieving high‐performance electrocatalysts.^[^ ^14^ ^]^ Thus, many attempts have been made to increase the electrochemical surface area (ECSA) via engineering nanostructures.^[^ ^21^, ^22^ ^]^

Catalysts powder‐coated on conductive substrates is commonly used as electrodes in energy conversion systems. However, such electrodes suffer from several drawbacks: (1) poor adhesion at the catalyst/substrate interface, (2) high contact resistance at the interface, (3) high ohmic resistance of the thick catalyst layer, (4) low catalyst utilization with buried active sites in the catalyst layer, and (5) a time‐consuming fabrication process with multiple steps.^[^ ^23^, ^24^ ^]^ Self‐supported electrodes with electrocatalysts grown in situ on conductive substrates have received much attention as a solution to these problems.^[^ ^23^, ^24^ ^]^ The seamless contact between the electrocatalyst and substrate without a binder minimizes the ohmic resistance, as well as facilitates fast electron transfer at the interface and high catalyst utilization. The seamless contact is also advantageous to improve the mechanical adhesion of electrocatalyst on substrate, which prevents the catalyst loss during the electrochemical reactions, resulting the improvement of catalytic stability.^[^ ^25^, ^26^, ^27^, ^28^, ^29^ ^]^ Moreover, one‐pot synthesis provides a facile fabrication method without post‐processing. In addition, when porous substrates are employed, they can be directly used as gas diffusion electrodes (GDEs) for advanced energy conversion systems using membrane electrode assemblies.^[^ ^30^ ^]^ Self‐supported electrodes can be prepared by several methods, such as hydro/solvothermal methods, vapor deposition, and electrodeposition.^[^ ^31^, ^32^ ^]^ Among them, the electrodeposition method provides several advantages to fabricate self‐supported electrodes, compared with other methods. Firstly, electrodeposition is a simple process that can be conducted in an aqueous solution under mild conditions within a short time, whereas the other preparation methods are usually conducted at high temperature and pressure, consuming huge extra energy.^[^ ^33^, ^34^, ^35^, ^36^ ^]^ Morphologies (e.g., particle, sphere, film, sheet, wire, foam, etc.) and composition of electrocatalysts can be easily modulated by controlling the deposition parameters such as electrolyte configuration, deposition potential/current, and deposition time. Moreover, this method is advantageous for electrode scale‐up and mass production, which are essential for the commercialization of energy conversion systems.^[^ ^37^ ^]^ However, due to the mild condition synthesis, this method often suffers to obtain elaborate control on crystallinity and composition of electrocatalysts. Nevertheless, the advantages of electrodeposition method facilitate its broad use in fabrication of self‐supported electrodes.

This review summarizes the recent developments in self‐supported electrodes prepared by electrodeposition for various energy conversion systems. Particularly, we focus on the cathodic reactions of several electrolyzers such as hydrogen evolution reaction (HER), electrochemical CO_2_ reduction (ECR), and electrochemical nitrogen reduction reaction (NRR). Firstly, we revisit the reported fundamentals of the cathodic reactions, and then we emphasize the advantages of the electrodeposition method for fabricating high‐performance electrodes.

### 1.1 HER

For decades, water electrolysis technology has been recognized as a promising energy conversion system that can produce high‐purity hydrogen without the emission of pollutants.^[^ ^38^, ^39^, ^40^ ^]^ The HER is the cathodic reaction of water electrolysis: the proton or water molecule is electrochemically reduced to hydrogen gas and its kinetics can be accelerated with a highly active electrocatalyst.^[^ ^41^, ^42^, ^43^ ^]^ Among pure metals, platinum exhibits superior catalytic activity because of its suitable hydrogen adsorption Gibbs free energy (Δ*G*_H*_).^[^ ^44^ ^]^ However, although various studies have been conducted to minimize the noble metal contents of electrocatalysts for HER,^[^ ^45^, ^46^, ^47^ ^]^ their high cost and scarcity have remained a big challenge. Thus, transition‐metal‐based catalysts for the HER have been in spotlight with their morphological and compositional control to enhance the catalytic activity.^[^ ^48^, ^49^, ^50^, ^51^ ^]^ Despite these efforts, insufficient HER activity inhibits their commercial application.

Understanding the HER mechanism is an important in designing an appropriate catalyst. The HER in acidic^[^ ^44^ ^]^ and alkaline media^[^ ^42^ ^]^ proceeds according to the mechanism summarized in Table 1.

**TABLE 1:** HER mechanism according to acidic and alkaline media

Reaction	Acid	Alkaline
Volmer	* + H^+^ + e^−^ → H*	* + H_2_O + e^−^ → H* + OH^−^
Heyrovsky	* + H^+^ + e^−^ + H* → H_2_ + *	* + H_2_O + e^−^ + H* → H_2_ + OH^−^ + *
Tafel	2H* → H_2_ + 2*	2H* → H_2_ + 2*
Overall	* + 2H^+^ + 2e^−^ → H_2_	* + 2H_2_O + 2e^−^ → H_2_ + OH^−^

* Active site on the surface of catalyst.

*Source*: Reproduced with permission.^[^ ^42^, ^44^ ^]^ Copyright 2015, Elsevier and Copyright 2012, American Chemical Society, respectively.

First, the Volmer reaction proceeds on the catalyst surface, where the proton or water molecule is reduced to form adsorbed H*. Subsequently, H_2_ can be generated by two different reaction pathways: the Heyrovsky reaction, in which the other proton or water in the electrolyte reacts with the adsorbed H*, or the Tafel reaction, in which the two H* adsorbed on the catalyst surface combine. The rate‐determining step (RDS) of the HER relies on the H binding ability of the catalyst surface, and a Pt catalyst with a suitable H binding strength exhibits superior intrinsic activity for the HER.^[^ ^53^ ^]^ For transition‐metal‐based catalysts, modulating the electronic structure by controlling the morphologies and compositions facilitates modifying Δ*G*_H__*_ to almost zero, which is effective in improving the HER activity.

### 1.2 ECR

ECR has in recent times been considered as an essential technology that can convert CO_2_ into valuable C_+_ hydrocarbon products.^[^ ^54^, ^55^ ^]^ Therefore, the ECR can contribute to decreasing the CO_2_ concentration in the atmosphere, thereby mitigating global warming. However, the CO_2_ molecule is thermodynamically stable, which requires a large overpotential to be electrochemically reduced, and the ECR is less favorable than HER in aqueous electrolytic systems.^[^ ^56^, ^57^ ^]^ As shown in Table 2, ECR involves a complex reaction mechanism, and various hydrocarbon compounds can be produced, inducing selectivity problems.^[^ ^16^ ^]^

**TABLE 2:** Standard electrode potentials of CO_2_ in aqueous solutions. Reproduced with permission.^[^ ^16^ ^]^ Copyright 2015, American Chemical Society

(1)	CO_2_ + 2H^+^ + 2e^−^ → CO + H_2_O	−0.52 V_NHE_
(2)	CO_2_ + 2H^+^ + 2e^−^ → HCOOH	−0.61 V_NHE_
(3)	CO_2_ + 8H^+^ + 8e^−^ → CH_4_ + 2H_2_O	−0.24 V_NHE_
(4)	2CO_2_ + 12H^+^ + 12e^−^ → C_2_H_4_ + 4H_2_O	−0.34 V_NHE_
(5)	2CO_2_ + 12H^+^ + 12e^−^ → C_2_H_5_OH + 3H_2_O	−0.32 V_NHE_
(6)	2H^+^ + 2e^−^ → H_2_	0 V_NHE_

Product selectivity is related to the binding and activation energies of intermediates adsorbed on the catalyst surface.^[^ ^58^ ^]^ For example, carbon monoxide can be selectively produced by the favorable formation of *COOH, whereas *OCHO is known as a key intermediate in formate production.^[^ ^59^ ^]^ For C_2_ products, lowering the kinetic barrier of C‐C coupling is an important factor in increasing selectivity.^[^ ^60^, ^61^ ^]^ Accordingly, various studies have been conducted to improve the catalytic properties by controlling the morphology, facet, grain boundary, composition, and oxidation state.

### 1.3 NRR

The electrochemical NRR has received much attention as a promising alternative to the Haber–Bosch process because it can produce NH_3_ without carbon emission under ambient conditions.^[^ ^62^, ^63^ ^]^ The overall NRR reaction mechanism depending on pH of electrolyte is as follows:^[^ ^64^, ^65^ ^]^

$$\begin{array}{ccc} & & N_{2}+8H^{+}+6e^{-}\to 2{{\mathrm{NH}}_{4}}^{+}\left(\text{in}\;\text{acidic}\;\text{media}\right)\\ & & N_{2}+8H_{2}O+6e^{-}\to 2{\mathrm{NH}}_{4}\mathrm{OH}+\;6{\mathrm{OH}}^{-}\left(\text{in}\;\text{alkaline}\;\text{media}\right) \end{array}$$

Although the NRR is thermodynamically favorable, a high potential barrier for the first electron and proton transfer shows slow kinetics.^[^ ^17^ ^]^ The mechanism of the electrochemical NRR can be categorized into two complicated pathways: dissociative and associative pathways.^[^ ^17^, ^66^, ^67^ ^]^ For the dissociative pathway, the cleavage of the N_2_ triple bond is initiated at the catalyst surface, and further hydrogenation occurs. Owing to the strong N_2_ triple bond, the dissociative pathway suffers from sluggish kinetics. On the contrary, in the associative pathway, the triple N_2_ bond is gradually cleaved by multiple hydrogenation steps, and the associative pathway is divided into alternating, distal, and enzymatic pathways.^[^ ^17^, ^66^, ^67^ ^]^ In addition, the competition with HER is a critical challenge facing electrochemical NRR owing to the narrow gap in the potential window between the NRR and HER. Furthermore, the low solubility of N_2_ gas in aqueous solution (0.66 mM under ambient conditions) leads to mass transfer limitations and hinders the catalytic activity.^[^ ^66^ ^]^ To solve these problems, many studies have been conducted on catalyst surface engineering to strengthen N_2_ adsorption and activation while minimizing the competing HER.

## 2 SELF‐SUPPORTED ELECTRODES PREPARED BY ELECTRODEPOSITION

### 2.1 Noble‐metal‐based electrodes for HER

#### 2.1.1 In acidic media

Noble‐metal‐based electrodes exhibit high catalytic activity for the acidic HER because neither strong nor weak binding strength of hydrogen on the catalyst surface provides the proper Δ*G*_H__*_ value.^[^ ^15^, ^44^, ^53^ ^]^ Thus, various strategies have been developed to reduce the amount^[^ ^68^, ^69^ ^]^ or to increase the catalyst utilization of noble metals using non‐noble elements.^[^ ^70^, ^71^, ^72^ ^]^ Table 3 summarizes the deposition conditions, noble metal loading amount, and HER activity of state‐of‐the‐art noble‐metal‐based electrodes in acidic media.^[^ ^68^, ^69^, ^70^, ^71^, ^72^ ^]^

**TABLE 3:** Summary of deposition conditions and HER performance of noble‐metal‐based electrodes in acidic media

		Deposition condition							Overpotential (mV)						
Catalyst	Loading mass	Electrolyte	Potential/current/time	Substrate	Temperature (℃)	Post‐treatment	Electrolyte	Tafel slope (mV dec^−1^)	η_10_	η_20_	η_50_	Exchange current density (mA cm^−2^)	Stability	Single‐cell test (Y/N)	Ref.
Pt300/CP	0.021 mg_Pt_ cm^−2^	3 mM K_2_PtCl_4_ + 0.5 M NaCl	Pulse: (0.4 V_SCE_ for 2 s) + (−0.9 V_SCE_ for 10 s) + (0.4 V_SCE_ for 3 s), 300 cycles	Carbon paper	RT	–	0.5 M H_2_SO_4_	36	45	–	–	0.56	–	Y	^[^ ^68^ ^]^
Pt30/C/CP	–	10 mM K_2_PtCl_4_ + 500 mM NaCl	Pulse: (0.4 V_SCE_ for 2 s) + (−0.9 V_SCE_ for 100 s) + (0.4 V_SCE_ for 3 s), 30 cycles	Carbon paper	RT	–	0.5 M H_2_SO_4_	–		–	–	–	–	N	^[^ ^69^ ^]^
Pt_1.8_MoS_2_	–	10 mM (NH_4_)_2_MoS_2_ + 0.2 M KCl + 6.4 mM Pt precursor	−1.3 V_Ag/AgCl_ for 10 min	Glassy carbon	RT	–	0.5 M H_2_SO_4_	48	80	–	–	–	CV for 1000 cycles	N	^[^ ^70^ ^]^
CoRe/CP	1.64 mg_Re_ cm^−2^	10 mM CoSO_4_·7H_2_O + 0∼5 mM NH_4_ReO_4_ + 100 mM Na_3_C_6_H_5_O_7_ + (NH_4_)_2_SO_4_	−1.5 V_SCE_ for 600 s	Carbon paper	RT	–	0.5 M H_2_SO_4_	63.6	45.1	–	–	–	−50 mA cm^−2^ for 10 h	N	^[^ ^71^ ^]^
Rh‐P10/CP‐0.40	72.6 μg_Rh_ cm^−2^	10 mM Na_3_RhCl_6_ + 5 mM NaH_2_PO_2_·6H_2_O	−0.4 V_SCE_ for 10 s	Carbon paper	RT	–	0.5 M H_2_SO_4_	28	23.1	–	–	–	−50 mA cm^−2^ for 10 h	N	^[^ ^72^ ^]^

Kim et al. fabricated an extremely low‐loading Pt catalyst on carbon paper (CP) by direct self‐terminated electrodeposition (SED),^[^ ^68^ ^]^ which can quench Pt electrodeposition passivated by H adsorption at an appropriate negative potential.^[^ ^73^, ^74^, ^75^ ^]^ By applying a repeated potential pulse number from 1 to 500, precise control of the Pt loading mass could be achieved in the submicrogram to submilligram range. Although the Pt deposits were invisible in field‐emission scanning electron microscopy (FESEM) images until a pulse number of 100, the intensity of the Pt 4f peaks obtained by X‐ray photoelectron spectroscopy (XPS) gradually increased with increasing pulse number. Among them, Pt/CP with a pulse number of 300 exhibited the highest HER activity with an overpotential of 45 mV at −10 mA cm^−2^ and a Tafel slope of 36 mV dec^−1^ in 0.5 M H_2_SO_4_ electrolyte. Furthermore, Pt/CP (Pt: 0.021 mg_Pt_ cm^−2^) electrodes were employed as a cathode in a proton exchange membrane water electrolyzer (PEMWE) single cell. The optimized catalyst demonstrated remarkable performance in terms of high mass activity, indicating high catalyst utilization in self‐supported electrodes prepared by electrodeposition. Further enhancement of the electrode was achieved using a C‐coated CP substrate to increase the ECSA.^[^ ^69^ ^]^ The repeated potential pulse facilitated the gradual increase in the ECSA and HER activity of Pt/C/CP, and they were then saturated over 50 pulses. The optimized Pt/C/CP electrode with 30 pulses showed higher HER current density than commercial Pt foil in 0.5 M H_2_SO_4_ electrolyte.

**FIGURE 1:**
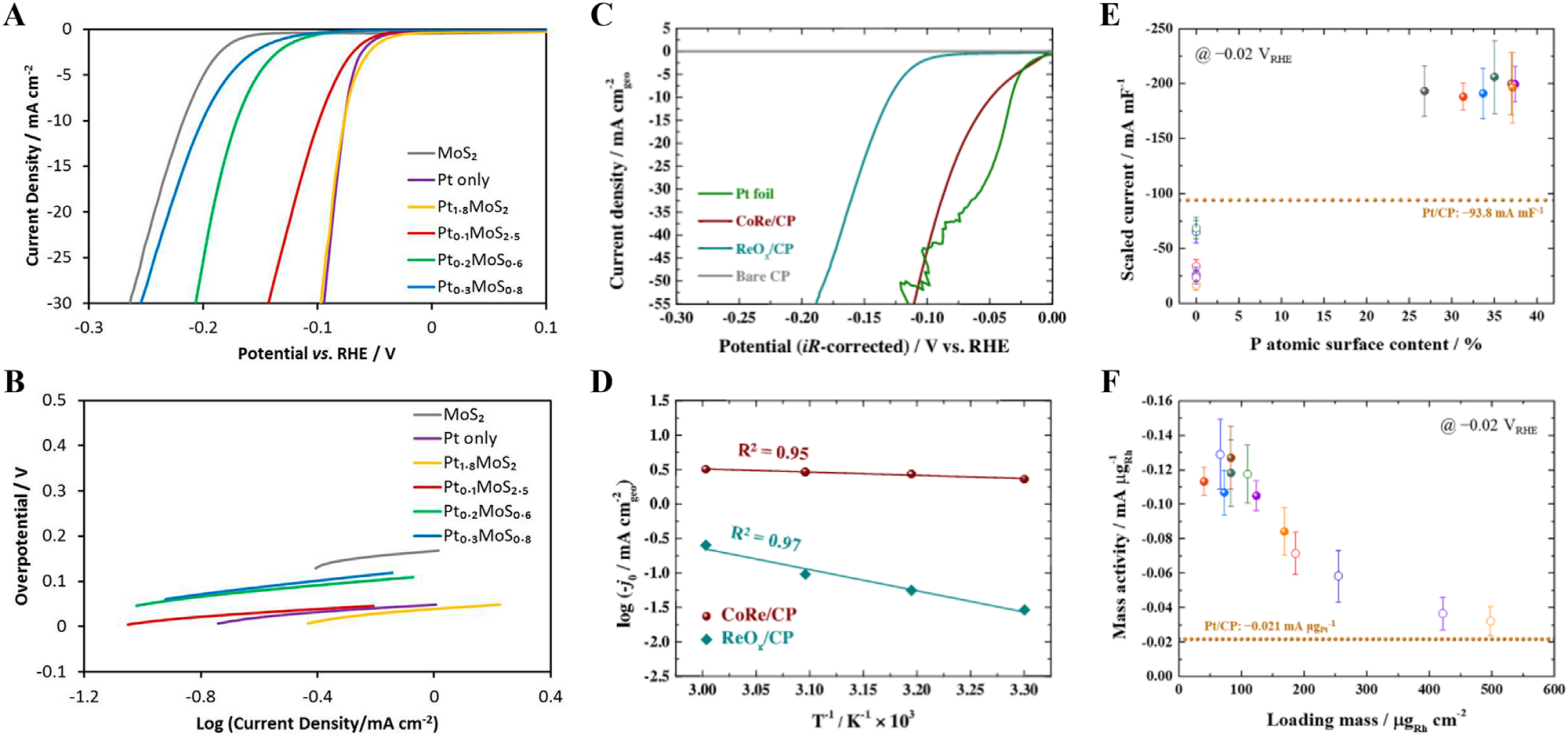
Self‐supported noble metal‐based electrodes for HER in acidic medium. (A) Polarization curves of the MoS_2_, Pt, and Pt‐MoS*_x_* composites in 0.5 M H_2_SO_4_. (B) Tafel plots for polarization curves in (A). Reproduced with permission.^[^ ^70^ ^]^ Copyright 2018, American Chemical Society. (C) Polarization curves of the CoRe/CP, ReO*_x_*/CP, and Pt foil in 0.5 M H_2_SO_4_. (D) Arrhenius plot for the acidic HER as a function of temperature. Reproduced with permission.^[^ ^71^ ^]^ Copyright 2021, Elsevier. (E,F) Scaled current and mass activity of the Rh/CP and Rh‐P/CP at −0.02 V_RHE_, respectively. Reproduced with permission.^[^ ^72^ ^]^ Copyright 2019, American Chemical Society

Along with non‐noble elements, the PGM catalysts exhibited a synergetic effect on HER activity.^[^ ^70^, ^71^, ^72^ ^]^ Chia et al. reported a Pt‐MoS*_x_* hybrid catalyst fabricated by electrodeposition.^[^ ^70^ ^]^ At a constant potential of −1.3 V_Ag/AgCl_, the composition of the hybrid catalysts was varied by controlling the Pt precursor concentration from 2.6 to 6.4 mM in the electrodeposition bath, which showed a significant effect on the elemental composition of the Pt‐MoS*_x_* hybrid catalyst, resulting in catalytic properties. All the Pt‐MoS*_x_* hybrid catalysts demonstrated a lower HER overpotential and Tafel slope than MoS_2_ (Figure 1A,B). Among them, Pt_1.8_MoS_2_ and Pt_0.1_MoS_2.5_ showed HER kinetics comparable to those of electrodeposited Pt. From the Tafel slope, it was revealed that the RDS in the HER mechanism of all the Pt‐MoS*_x_* catalysts was the Heyrovsky desorption, whereas the Volmer adsorption limited the HER on electrodeposited MoS_2_. Kim et al. reported a CoRe alloy catalyst for CP by electrodeposition.^[^ ^71^ ^]^ By modulating the electrodeposition parameters, the Re composition and loading mass were controlled. The optimal CoRe/CP electrode showed a spherical morphology with a diameter of 5 μm and Re content of 37.0%. Compared with the ReO*_x_*/CP electrodeposited without the Co precursor, the optimized CoRe/CP electrode exhibited higher HER activity with an overpotential of 45.1 mV at −10 mA cm^−2^ (Figure 1C). The CoRe/CP exhibited a much lower HER activation energy (8.99 kJ mol^−1^ K^−1^) than ReO*_x_*/CP (58.87 kJ mol^−1^ K^−1^) for (Figure 1D). In addition, stability tests were conducted at −50 mA cm^−2^ for 10 h, resulting in a potential increase at the initial stage due to Co dissolution, as confirmed by EDS analysis. Electrodeposited Rh‐P catalysts were also effective in HER.^[^ ^72^ ^]^ First, the deposition parameters for the Rh/CP electrode were optimized by varying the electrodeposition time to control the Rh loading mass and morphology. At a certain deposition time, the HER intrinsic activity was maximized owing to the highly exposed Rh(111) facet. To enhance the HER activity, a 5 mM P precursor was added to the electrodeposition bath. With deposition potential increased, the particle size and coverage gradually increased with agglomeration. The Rh‐P catalyst on CP (Rh‐P/CP) has smaller particle size than Rh/CP. The P atomic surface concentration was controlled in the range of 27–37%. All the Rh‐P/CP electrodes exhibited higher HER intrinsic activity than the Rh/CP electrode (Figure 1E). Furthermore, the mass activity of all the Rh/CP and Rh‐P/CP electrodes surpassed that of the Pt/CP electrode, as shown in Figure 1F. The stability of the Rh‐P10/CP‐0.40 which demonstrates the highest HER intrinsic activity was confirmed at −50 mA cm^−2^ for 10 h.

#### 2.1.2 In alkaline media

Noble‐metal‐based electrodes also show superior catalytic activity for the HER in alkaline media.^[^ ^76^, ^77^, ^78^, ^79^, ^80^, ^81^ ^]^ However, the HER activity of noble metals (e.g., Pt) in alkaline media is 2–3 orders of magnitude lower than that in acidic media, owing to the high energy barrier for the water dissociation step,^[^ ^82^ ^]^ which remains a challenge. Table 4 summarizes the deposition conditions and HER activity of state‐of‐the‐art noble‐metal‐based electrodes in an alkaline media.^[^ ^76^, ^77^, ^83^, ^84^, ^85^, ^86^, ^87^, ^88^, ^89^, ^90^, ^91^, ^92^ ^]^

**TABLE 4:** Summary of deposition conditions and HER performance of noble metal‐based electrodes in alkaline media

		Deposition condition							Overpotential (mV)						
Catalyst	Loading mass	Electrolyte	Potential/current/time	Substrate	Temperature (℃)	Post‐treatment	Electrolyte	Tafel slope (mV dec^−1^)	η_10_	η_20_	η_50_	Exchange current density (mA cm^−2^)	Stability	Single‐cell test (Y/N)	Ref.
450‐nm Pt nanopillar‐array	–	5 mM H_2_PtCl_6_	−0.1 mA cm^−2^ for 8 min	AAO	50	Solution treatment	1.0 M KOH	59	78	120	265	–	–	N	^[^ ^76^ ^]^
PtPd@NLS	–	1 mM H_2_PtCl_6_ + 1 mM PdCl_2_ + 0.2 M H_2_SO_4_	−0.2 V for 10 s	NLS modified GCE	–	–	1.0 M KOH	124	46	77	113	–	−30 mV for 12 h / CV for 1000 cycles	N	^[^ ^77^ ^]^
Pt_SA_‐NiO/Ni	–	0.05 mM H_2_PtCl_6_ + 1.0 M KOH	CV: 0 ∼ −0.5 V_RHE_, 200 cycles, 50 mV s^−1^	NiO/Ni@Ag NWs	–	–	1.0 M KOH	27.1	26	38	63	–	−20 mA cm^−2^ for 30 h/ CV for 1000 cycles	N	^[^ ^83^ ^]^
Pt‐Co(OH)_2_/CP	–	20 mM H_2_PtCl_6_·6H_2_O + 0.5 mM NaH_2_PO_2_ + 11.7 mg C_6_H_5_Na_3_O_7_·6H_2_O	−0.25 mA cm^−2^ for 15 min	Co(OH)_2_/CP	30	–	0.1 M KOH	58	37.6	52	98	–	−10 mA cm^−2^ for 20,000 s/ CV for 1000 cycles	N	^[^ ^84^ ^]^
Pt‐Co(OH)_2_/CC	6.9 mg_metal_ cm^−2^	20 mM H_2_PtCl_6_·6H_2_O + 0.5 mM NaH_2_PO_2_ + 11.7 mg C_6_H_5_Na_3_O_7_·6H_2_O	−0.25 mA cm^−2^ for 15 min	Co(OH)_2_/CC	30	–	1.0 M KOH	70	32	54	95	–	CV for 1000 cycles	N	^[^ ^85^ ^]^
Pt‐Ni NTAs	0.016 mg_Pt_ cm^−2^	4 mM H_2_PtCl_6_·6H_2_O + 0.5 M H_2_SO_4_	−0.25 V_Ag/AgCl_ for 60 s	Ni NTAs	–	–	1.0 M KOH	38	23	33	48	–	Current step at −10, −20, −50 mA cm^−2^ for total 144 h	N	^[^ ^86^ ^]^
Fe‐Pt film	29 μg	3.6 mM FeCl_2_·4H_2_O + 1.3 mM Na_2_PtCl_6_ + 5 g L^−1^ Pluronic P‐123	−1.1 V_Ag/AgCl_ for 600 s	Au	25	–	1.0 M KOH	–	74	106	163	–	CV for 100 cycles	N	^[^ ^87^ ^]^
Cu‐Ru/Ti	0.063 mg cm^−2^	K_2_RuCl_6_ + 1 M NaOH	−1.2 V_Ag/AgCl_ for 24 h	CuO NPls/Ti	–	–	1.0 M NaOH	34	23	43	68	–	−0.2 V_RHE_ for 100 h/Current step at −10, −50, −100 mA cm^−2^ for total 72 h/ CV for 1000 cycles	N	^[^ ^88^ ^]^
Pd/Co(OH)_2_/graphene/NF	–	9.6 mg PdCl_2_ + 0.375 g NH_4_Cl + 1.17 g EDTA + NH_4_OH	−10 mA cm^−2^ for 30 min	Co(OH)_2_/graphene/NF	–	–	1.0 M KOH	53	124	170	248	–	CV for 1000 cycles	N	^[^ ^89^ ^]^
Pt‐Ni_0.6_Co_0.4_ (HCO_3_)_2_/NF	3 mg_catal_ cm^−2^	0.3 mM H_2_PtCl_6_ + 0.05 M NaCl	−2.0 V_SCE_ for 5 min	Ni_0.6_Co_0.4_(HCO_3_)_2_/NF	–	–	1.0 M KOH	39	56	89	175	4.2	−10 mA cm^−2^ for 30 h	N	^[^ ^90^ ^]^
Pt@GO @Ni‐Cu@NF	–	3 mM H_2_PtCl_6_·6H_2_O + 0.5 M H_2_SO_4_	−0.4 V_SCE_ for 120 s	GO@Ni‐Cu@NF	–	–	0.1 M KOH	51	31	50	95	–	Current step at 10–00 mA cm^−2^ with a step of 10 mA cm^−2^ at 6000 s / CV for 1000 cycles	N	^[^ ^91^ ^]^
Pt/Ni_3_S_2_/NF	53 μg cm^−2^	20 mM H_2_PtCl_6_·6H_2_O + 0.5 mM NaH_2_PO_2_ + 11.7 mg C_6_H_5_Na_3_O_7_·6H_2_O	−0.25 mA cm^−2^ for 15 min	N_3_S_2_/NF	30	–	1.0 M KOH	73	10	38	–	5.43	−20 mA cm^−2^ for 20 h	N	^[^ ^92^ ^]^

Cheng et al. reported a Pt nanopillar‐array catalyst electrodeposited on an anodized aluminum oxide substrate, and the length of the Pt nanopillar could be controlled by adjusting the electrodeposition time.^[^ ^76^ ^]^ The Pt nanopillar array with a length of 450 nm exhibited an overpotential of 78 mV at −10 mA cm^−2^ and a Tafel slope of 59 mV dec^−1^ in a 1.0 M KOH electrolyte. It was revealed that the low charge transfer resistance of the 3D electrode and the high desorption capability of hydrogen bubbles at the nanotips improved the HER activity of the Pt nanopillar‐array. PtPd nanoparticles (NPs) prepared by co‐electrodeposition on a nitrogenous loofah sponge (NLS) also showed high HER activity in alkaline electrolytes.^[^ ^77^ ^]^ The NLS fabricated by the carbonization of loofah sponge exhibited a wrinkled sheet structure, which prevented the aggregation of noble metal particles. The PtPd@NLS contained 6.95 wt% Pt and 8.65 wt% Pd. In 1.0 M KOH, the PtPd@NLS catalyst demonstrated an HER overpotential of 46 mV at −10 mA cm^−2^ and a Tafel slope of 124 mV dec^−1^. EIS analysis confirmed that the bimetallic properties of the PtPd alloy had a better electron transfer rate.

**FIGURE 2:**
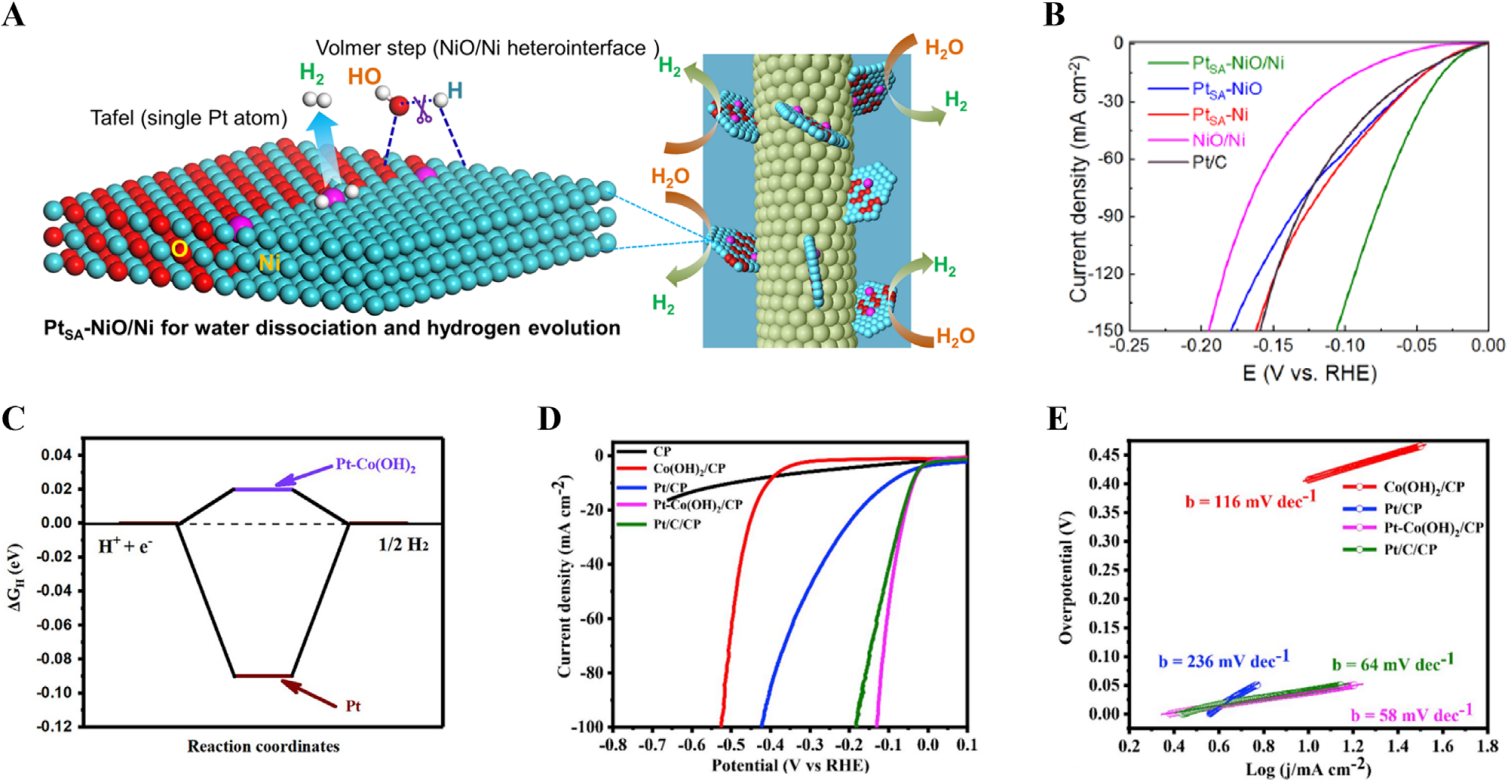
Self‐supported noble metal‐based electrodes for HER in alkaline medium. (A) HER mechanism of Pt_SA_‐NiO/Ni in alkaline media. (B) HER activity of Pt_SA_‐NiO/Ni, Pt_SA_‐NiO, Pt_SA_‐Ni, NiO/Ni, and Pt/C. Reproduced with permission.^[^ ^83^ ^]^ Copyright 2021, Springer Nature. (C) DFT calculation of hydrogen adsorption free energy for Pt and Pt‐Co(OH)_2_ catalyst. (D,E) Polarization curves and Tafel plots of as‐prepared electrocatalyst in 0.1 M KOH. Reproduced with permission.^[^ ^84^ ^]^ Copyright 2019, Elsevier

Recently, heterostructured catalysts with noble metal/transition metal combinations have been considered as effective materials for accelerating the water dissociation step, owing to their moderate hydrogen adsorption energy.^[^ ^83^, ^84^, ^85^, ^86^, ^87^, ^88^, ^89^, ^90^, ^91^, ^92^ ^]^ Zhou et al. reported single‐atom Pt immobilized on NiO/Ni, which enables the modulation of the adsorption affinity toward both OH* and H*, resulting in a faster water dissociation step and a reduced energy barrier for the Volmer step (Figure 2A).^[^ ^83^ ^]^ Furthermore, the anchored Pt atoms on the NiO/Ni interfaces induced a more suitable H binding energy (Δ*G*_H*_: −0.07 eV) than that of Pt atoms in NiO (Δ*G*_H*_: 0.74 eV) and Ni (Δ*G*_H*_: −0.38 eV), which efficiently facilitated H* conversion and H_2_ desorption for improved HER activity in alkaline media. The electrode exhibited superior HER performance with an overpotential of 26 mV at −10 mA cm^−2^ and a mass activity of −20.6 A mg^−1^ at an overpotential of 100 mV (Figure 2B). The combination of Pt and Co(OH)_2_ can also improve the water dissociation step in alkaline media.^[^ ^84^, ^85^ ^]^ Based on density functional theory (DFT) calculations, which reveal suitable hydrogen binding energy on Pt‐Co(OH)_2_ (−0.29 eV), two‐step galvanostatic electrodeposition was conducted to fabricate Pt‐Co(OH)_2_/CP (Figure 2C).^[^ ^84^ ^]^ The Pt‐Co(OH)_2_/CP electrode showed HER performance with an overpotential of 37.6 mV at −10 mA cm^−2^, which was better than that of the Pt/CP electrode (Figure 2D). Meanwhile, Xing et al. prepared Pt NPs electrodeposited on Co(OH)_2_ nanosheets hydrothermally fabricated on a carbon cloth (CC).^[^ ^85^ ^]^ The CC‐supported Co(OH)_2_ nanosheets provided a large surface area for Pt electrodeposition. ICP‐MS analysis confirmed a low Pt content (5.7 wt%). The Pt‐Co(OH)_2_/CC electrode showed an overpotential of 32 mV at −10 mA cm^−2^, which was better than that of the commercial Pt/C/CC electrode. Nairan et al. conducted Pt electrodeposition on a Ni nanothorn array (NTA) fabricated on Ti foil via a modified magnetic‐field‐driven growth process.^[^ ^86^ ^]^ The nanowire morphology of Pt‐Ni NTAs accumulated H^+^ near the catalyst surface in a “pseudoacidic” local environment, which facilitated H^+^ adsorption on the active site. From the DFT calculations, the NT structure with a large surface area could promote the induced electric field on the tips and demonstrated eight times higher H^+^ adsorption than a smooth NW surface. The strong local electric field around the NT structure facilitated faster H^+^ mass transfer toward the catalyst surface and reaction kinetics for the HER in alkaline media. Furthermore, ICP‐MS analysis confirmed that Pt‐Ni NTA had low Pt loading mass (0.016 mg_Pt_ cm^−2^). As a result, the Pt‐Ni NTA electrode showed a small overpotential of 23 mV at −10 mA cm^−2^ and stable performance during stability test at a current step of −10, −50, −100 mA cm^−2^ for total 144 h. Micelle‐assisted electrodeposition enabled the fabrication of Fe‐Pt alloy films on different metallic seed layers with varying Fe/Pt ratios.^[^ ^87^ ^]^ Fe‐Pt alloy films on Au seed layers showed an ultrasmooth morphology with nanoscale pores and narrow cracks. ICP‐OES analysis confirmed that a large amount of Fe (21 wt%) was obtained. Compared to the mesoporous Pt catalyst, the mesoporous Fe‐rich Fe‐Pt alloy film exhibited better HER performance in an alkaline media with an overpotential of 74 mV at −10 mA cm^−2^. They demonstrated that the large surface‐area‐to‐volume ratio and exposure of a large fraction of active Pt centers improved the HER activity of the Fe‐Pt alloy film.

### 2.2 Non‐noble‐metal‐based electrodes for HER

#### 2.2.1 In acidic media

Although noble‐metal‐based electrodes show high HER activity, their high price and scarcity inhibit the commercialization of water electrolysis systems. Accordingly, transition‐metal‐based electrodes have been investigated by modulating the catalytic properties to enhance the HER performance.^[^ ^93^, ^94^, ^95^, ^96^, ^97^, ^98^, ^99^, ^100^ ^]^ For decades, various studies on the fabrication of transition metal compounds such as phosphides^[^ ^101^, ^102^, ^103^, ^104^, ^105^, ^106^, ^107^, ^108^, ^109^ ^]^ and sulfides^[^ ^110^, ^111^, ^112^, ^113^ ^]^ have been conducted. The synergetic effect between transition metals and nonmetals can contribute to enhancing catalytic activity. The relatively high electronegativity of nonmetals modulates the electronic structure of the catalyst, resulting in a hydrogen adsorption free energy approaching zero. Table 5 summarizes the deposition conditions, catalyst loading amount, and HER activity of state‐of‐the‐art transition metal compounds in acidic media.^[^ ^93^, ^94^, ^95^, ^96^, ^97^, ^98^, ^99^, ^100^, ^101^, ^102^, ^103^, ^104^, ^105^, ^106^, ^107^, ^108^, ^109^, ^110^, ^111^, ^112^, ^113^ ^]^

**TABLE 5:** Summary of deposition conditions and HER performance of non‐noble‐metal‐based electrodes in acidic media

		Deposition condition							Overpotential (mV)						
Catalyst	Loading mass	Electrolyte	Potential/current/time	Substrate	Temperature (℃)	Post‐treatment	Electrolyte	Tafel slope (mV dec^−1^)	η_10_	η_20_	η_50_	Exchange current density (mA cm^−2^)	Stability	Single‐cell test (Y/N)	Ref.
NiW	–	45 mM H_2_WO_4_ + 60 mM Na_2_SO_3_ + 52 mM Na_3_C_6_H_5_O_7_ + 125 mM NiSO_4_·6H_2_O	−1.2 V_SCE_ for 300 s	FTO	RT	–	0.5 M H_2_SO_4_	122	205	–	–	–	–	N	^[^ ^93^ ^]^
Cu_99.2_Mo_0.8_	0.06 mg_catal_ cm^−2^	0.05 M CuSO_4_·7H_2_O + 0.01 M Na_2_MoO_4_·2H_2_O + 0.25 M Na_3_C_6_H_5_O_7_	−1.0 V_SCE_ for 600 s	Ti foil	RT	–	0.5 M H_2_SO_4_	101	–	–	–	–	CV for 500 cycle	Y	^[^ ^94^ ^]^
Cu_44.4_Ni_46_Mo_9.6_	1.33 mg_catal_ cm^−2^	0.03 M CuSO_4_·5H_2_O + 0.1 M Na_2_MoO_4_·2H_2_O + 0.3 M NiSO_4_·6H_2_O + C_6_H_5_Na_3_O_7_·2H_2_O	−1.5 V_SCE_ for 600 s	Carbon paper	RT	–	0.5 M H_2_SO_4_	27.7	18	–	70	2.24	−10 mA cm^−2^ for 24 h / CV for 5000 cycle	Y	^[^ ^95^ ^]^
Co_59_Cu_41_	–	0.50 M CoSO_4_·7H_2_O + 0.15 M CuSO_4_·5H_2_O + 0.25 M Na_3_C_6_H_5_O_7_	−1.2 V_SCE_ for 300 s	Ti foil	RT	–	0.5 M H_2_SO_4_	103	342	–	–	–	−10 mA cm^−2^ for 12 h	Y	^[^ ^96^ ^]^
NiMo/CF/CP	–	0.3 M NiSO_4_·6H_2_O + 0.2 M Na_2_MoO_4_·2H_2_O + 0.3 M C_6_H_5_Na_3_O_7_·2H_2_O + 0.03 M NaOH	−1.4 V_SCE_ for 300 s	Cu foam on CP	RT	–	0.5 M H_2_SO_4_	96	68.7	–	–	–	–	Y	^[^ ^97^ ^]^
Ni_96_W_4_/ Cu NW/CP	–	0.13 M NiSO_4_·6H_2_O + 0.12 M Na_2_WO_4_·2H_2_O + 0.25 M Na_3_C_6_H_5_O_7_ + 0.1 M H_2_SO_4_	−1.6 V_SCE_ for 600 s	Cu nanowire on CP	RT	–	0.5 M H_2_SO_4_	40	56	79	181	–	−10 mA cm^−2^ for 12 h	Y	^[^ ^9^ ^8]^
MoO_2_@CoMo/ CP	∼6 mg_catal_ cm^−2^	300 mM CoSO_4_·7H_2_O + 50 mM Na_2_MoO_4_·4H_2_O + 30 mM NaOH + 300 mM C_6_H_5_Na_3_O_7_·2H_2_O	−2.0 V_SCE_ for 900 s	Carbon paper	RT	Electrochemical etching	0.5 M H_2_SO_4_	50.9	–	–	76.0	–	−10 mA cm^−2^ for 30 h −50 mA cm^−2^ for 30 h	N	^[^ ^99^ ^]^
E‐NiMo‐7.5@0.02/CP	0.88 mg_catal_ cm^−2^	10 mM Na_2_MoO_4_·2H_2_O + 100 mM C_6_H_5_Na_3_O_7_·2H_2_O + 1000 mM NiSO_4_·6H_2_O	−1.0 V_SCE_ for 1800 s	Carbon paper	RT	Electrochemical etching	0.5 M H_2_SO_4_	25.3	27.9	–	–	–	−10 mA cm^−2^ for 24 h	N	^[^ ^100^ ^]^
MoS*_x_*/CP	–	10 mM (NH_4_)_2_MoS_4_ + 0.2 M NaOH + 0.5 M NaClO_4_·H_2_O	−1.0 V_SCE_ for 300 s	Carbon paper	RT	–	0.5 M H_2_SO_4_	42.3	159.8	–	–	–	–	Y	^[^ ^110^ ^]^
Ni_0.96_Mo_0.04_S	0.10 mg_catal_ cm^−2^	0.05 M NiCl_2_·6H_2_O + Na_2_MoO_4_·2H_2_O (0.04 mole ratio of Mo to Ni) + 1 M thiourea	CV: −1.0 ∼ 0.4 V_Ag/AgCl_, 25 cycles, 5 mV s^−1^	FTO	RT	–	0.5 M H_2_SO_4_	50	180	–	–	–	−200 mV_RHE_ for 24 h	N	^[^ ^111^ ^]^
CN/CNL/MoS_2_/CP	–	1.2 g of NiCl_2_·6H_2_O + 1.2 g of CoCl_2_·6H_2_O	−1.0 V_Ag/AgCl_ for 30 s	MoS_2_/CP	RT	Annealing	0.5 M H_2_SO_4_	77	112	–	–	–	–	N	^[^ ^112^ ^]^
Ni_0.64_Co_0.36_O*_x_* S_0.14_/CP	–	0.06 M NiCl_2_·6H_2_O + 0.04 M CoCl_2_·6H_2_O + 0.5 M H_3_BO_3_	1step: −3 mA cm^−2^ for 300 s 2step: −55 mA cm^−2^ for 300 s	Carbon paper	RT	Sulfur ion exchange	0.5 M H_2_SO_4_	78.0	172	–	–	0.064	–	Y	^[^ ^113^ ^]^
FePNRs/VAGNs/CC	0.776 mg_catal_ cm^−2^	0.05 M FeSO_4_·7H_2_O	−1.6 V_Ag/AgCl_ for 20 min	Carbon cloth	RT	Phosphidation	0.5 M H_2_SO_4_	42	53	–	–	0.275	−20 mA cm^−2^ for 20 h / CV for 5000 cycle	N	^[^ ^101^ ^]^
Fe_2_P@rGO	0.47 mg_catal_ cm^−2^	0.05 M FeSO_4_ + 1.0 M Na_2_S_2_O_3_ aqueous solution at a volume ratio of 1:4 + GO suspension at molar concentration ratios of 8:2	CV: −1.4 ∼ 0.6 V_SCE_, 20 cycles, 50 mV s^−1^	Ti plate	RT	Phosphidation	0.5 M H_2_SO_4_	55.2	101	–	–	0.146	−125 mV for 12 h	N	^[^ ^102^ ^]^
Ni_78_P_22_	–	0.5 M NiCl_2_·6H_2_O + 0.5 M NaH_2_PO_2_·H_2_O + 0.1 M NH_4_Cl	1 pulse : −1.0 V_SCE_ for 10 ms and −1.8 ∼ −1.0 V_SCE_ for 20 ms	Ti foil	RT	–	0.5 M H_2_SO_4_	38	105	–	–	–	–	Y	^[^ ^103^ ^]^
FeP	2.6 mg_catal_ cm^−2^	5.0 g FeSO_4_·6H_2_O + 1.58 g NaH_2_PO_2_ and 0.25 ml of formic acid in ultra pure water to reach final volume of 25 ml. pH to 1.5 adding H_2_SO_4_	1step: −1.7 V for 120 s 2step: −0.1 V for 240 s	Cu foil	RT	–	0.5 M H_2_SO_4_	55	66	–	–	–	−10 mA cm^−2^ for 24 h	N	^[^ ^104^ ^]^
Ni−Cu−P	21 mg_catal_ cm^−2^	0.2 M NiCl_2_·6H_2_O + 0.2 M NaH_2_PO_4_·H_2_O + 0.25 M NH_4_Cl + 0–0.04 M CuCl_2_·2H_2_O	−10 mA for 1200 s	Ni sheet	RT	–	0.5 M H_2_SO_4_	69	150	–	–	–	–	N	^[^ ^105^ ^]^
FeCoP/CF	21 mg_catal_ cm^−2^	250 mM FeSO_4_ ·7H_2_O + 250 mM CoSO_4_ ·7H_2_O + 100 mM NaH_2_PO_2_ ·H_2_O + 100 mM C_6_H_8_O_7_ + 50 mM (NH_4_)_2_SO_4_	−1.2 V_SCE_ for 600 s	Carbon paper	RT	–	0.5 M H_2_SO_4_	136	125	–	–	6× 10^−5^	−175 mV for 50 h	Y	^[^ ^106^ ^]^
Co_59_P_20_B_21_/CP	–	1 mM CoCl_2_·6H_2_O + 0.1 M NaCl + 250 mM NaH_2_PO_2_·H_2_O + 250 mM H_3_BO_4_	−1.5 V_SCE_ for 600 s	Carbon paper	RT	–	0.5 M H_2_SO_4_	68	172	–	–	0.031	–	N	^[^ ^107^ ^]^
Co−P−0.3	–	0.5 M CoCl_2_·6H_2_O + 0.5 M NaPO_2_H_2_·H_2_O + 0.1 M NH_4_Cl	−1.0 V_SCE_ for 20 ms −0.3 V_SCE_ for 10 ms with total charge of 20 C cm^−2^	Carbon paper	RT	–	0.5 M H_2_SO_4_	60.1	143.8	–	–	–	–	Y	^[^ ^108^]
L−Ni_0.46_P_0.54_/CP	–	100−250 mM NiCl_2_·6H_2_O + 500 mM NaPO_2_H_2_·H_2_O + 100 mM NaCl + 100 mM Na_3_C_6_H_5_O_7_	−1.5 V_SCE_ for 600 s	Carbon paper	RT	Electrochemical etching	0.5 M H_2_SO_4_	53.4	103	–	–	0.117	−50 mA cm^−2^ for 10 h	Y	^[^ ^109^ ^]^

**FIGURE 3:**
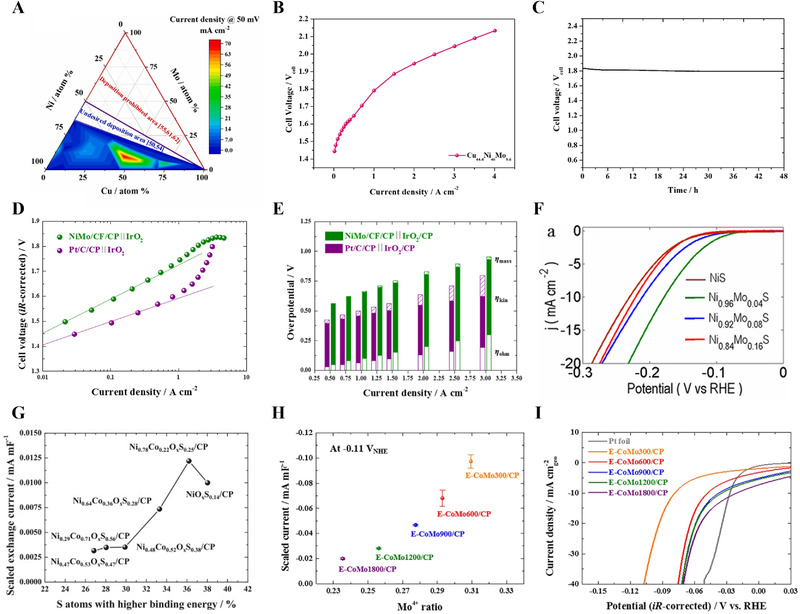
Self‐supported non noble metal‐based electrodes for HER in acidic medium. (A) Contour plot of the geometric HER activity of the CuNiMo ternary catalysts. (B) *V*–*J* curve for PEMWE with Cu_44.4_Ni_46_Mo_9.6_ cathode. (C) Durability test of PEMWE single cell at 1 A/cm^2^ for 48 h. Reproduced with permission.^[^ ^95^ ^]^ Copyright 2021, Elsevier. (D) Tafel plot of polarization curves for PEMWE single cell with NiMo/CF/CP and Pt/C/CP. (E) Overpotential analysis of single cell test in (D). Reproduced with permission.^[^ ^97^ ^]^ Copyright 2019, Elsevier. (F) Polarization curves of NiS and Ni_1−_ *_x_* Mo*_x_* S in 0.5 M H_2_SO_4_. Reproduced with permission.^[^ ^111^ ^]^ Copyright 2017, American Chemical Society. (G) Scaled exchange current of the Ni*_y_* Co_1−_ *_y_* O*_x_*/CP as a function of S atoms with higher binding energy. Reproduced with permission.^[^ ^113^ ^]^ Copyright 2018, Elsevier. (H) Scaled current of the E‐CoMo#/CP depending on the Mo^4+^ ratio. (I) Polarization curves of the Pt foil and E‐CoMo#/CP. Reproduced with permission.^[^ ^99^ ^]^ Copyright 2020, Elsevier

It has been reported that bimetallic electrodes are effective in improving HER activity, depending on their components and compositions. Mollamahale et al. fabricated NiW on fluorine‐doped tin oxide (FTO) by electrodeposition at different potentials for 300 s.^[^ ^93^ ^]^ The morphology of Ni‐W exhibited spherical NP shapes with a size of 100–200 nm. The atomic ratio of W to Ni was 1:100, and W was incorporated into the Ni structure. Compared with Ni, all Ni‐W NP electrodes showed higher HER activity. The Ni‐W@−1.2 V exhibited the highest activity with an overpotential of 205 mV at −10 mA cm^−2^ and a Tafel slope of 122 mV dec^−1^ in 0.5 M H_2_SO_4_ electrolyte. Similarly, Cu*_x_* Mo_100−_ *_x_* alloys were electrodeposited on Ti foil.^[^ ^94^ ^]^ The Mo composition was controlled by varying the Mo precursor concentration from 0.01 to 0.60 M in the electrolyte. Compared with the Cu_100_ electrode, all the Cu*_x_* Mo_100−_ *_x_* electrodes showed high HER activity, and Cu_96.2_Mo_3.8_ demonstrated the highest geometric current density of −88.9 mA cm^−2^ at −0.50 V_RHE_ with a Tafel slope of 101 mV dec^−1^ in 0.5 M H_2_SO_4_ electrolyte. In addition, the intrinsic HER activity was obtained by normalizing the geometric current density using the ECSA. The intrinsic activity of the Cu_99.2_Mo_0.8_ electrode was 7.4 times higher than that of the Cu_100_ electrode at −0.50 V_RHE_, indicating a strong synergistic effect with addition of a small amount of Mo. The performance of a PEMWE single cell with a Cu_96.2_Mo_3.8_/CP cathode demonstrated a current density of 0.5 A cm^−2^ at 1.90 V_cell_. Kim et al. fabricated Co‐Cu alloys on Ti foil by electrodeposition at −1.2 V_SCE_ for 300 s.^[^ ^96^ ^]^ The bulk composition of the Co‐Cu alloy was controlled by varying the metal precursor concentration. With an increase in the Cu content, the morphology gradually changed from needle‐shaped particles to small round particles, whereas the hexagonal close‐packed structure of Co_100_ changed to a face‐centered cubic structure. The HER activity was maximized at a Cu content of 41%. Optimized deposition conditions were used on the CP substrate. The Co_54_Cu_46_/CP cathode demonstrated a current density of 1.2 A cm^−2^ at 2.0 V_cell_ in a PEMWE single‐cell test. In the same group, CuNiMo ternary catalysts were electrodeposited on a CP substrate using chronoamperometry (CA).^[^ ^95^ ^]^ The ternary Cu_44.4_Ni_46.0_Mo_9.6_ alloy catalyst showed higher HER activity than single and binary catalysts, with a low overpotential of 18 mV at −10 mA cm^−2^ and a Tafel slope of 27.7 mV dec^−1^ in 0.5 M H_2_SO_4_ electrolyte (Figure 3A). Although the intrinsic activity slightly decreased after Cu introduction, the overall catalytic performance, including stability, was highly improved. Furthermore, a PEMWE single‐cell test with the Cu_44.4_Ni_46_Mo_9.6_/CP cathode demonstrated high performance with a current density of 1.62 A cm^−2^ and excellent stability at 1.9 V_cell_ for 48 h (Figure 3B,C).

Another strategy to increase the ECSA is to use a roughened metallic support.^[^ ^97^ ^]^ Kim et al. conducted a two‐step electrodeposition method: (1) bubble‐templating electrodeposition was used to prepare Cu foam (CF) on CP and (2) NiMo was directly electrodeposited on CF/CP. The optimized NiMo/CF/CP showed a low HER overpotential of 68.7 mV at −10 mA cm^−2^ and a Tafel slope of 96.0 mV dec^−1^. A PEMWE single cell with NiMo/CF/CP cathode showed a remarkable current density of ∼2.0 A cm^−2^ at 2.0 V_cell_ (Figure 3D). By overpotential subdivision in Figure 3E, a lower mass‐transfer overpotential of NiMo/CF/CP than that of the Pt/C electrode was confirmed, indicating the porous structure is advantageous for improving the mass transfer in the high‐current‐density region. Furthermore, acceptable stability of the PEMWE single‐cell was obtained at a constant cell voltage of 2.0 V_cell_ for 36 h. Similarly, two‐step electrodeposition was employed to fabricate NiW catalysts supported by Cu nanowires (NWs) on CP.^[^ ^100^ ^]^ As a first step, Cu NWs were prepared on CP by electrodeposition and subsequent oxidation/reduction. By varying the metal precursor concentration, Ni*_x_* W_100‐_ *_x_* was electrodeposited on the prepared Cu NW/CP at −1.6 V_SCE_ for 600 s. The optimized amorphous Ni_96_W_4_/Cu NW/CP showed the lowest HER overpotential of 56.0 mV at −10 mA/cm^2^ with a Tafel slope of 40 mV/dec in 0.5 M H_2_SO_4_. The HER stability of Ni_96_W_4_/Cu NW/CP was evaluated over 1000 cycles and chronopotentiometry was performed at −10 mA cm^−2^ for 12 h. It was revealed that the activity decay was attributable to the further oxidation of Ni, NiO, and W, as confirmed by XPS analysis. Furthermore, the Ni_96_W_4_/Cu NW/CP was employed as a cathode for a PEMWE single cell, which demonstrated a current density of 1.79 A cm^−2^ at 2.0 V_cell_.

Electrodeposited transition metal phosphides are well‐known catalysts for the HER in acidic electrolytes. Kim et al. manufactured a Ni−P catalyst on a Ti foil through pulse electrodeposition.^[^ ^103^ ^]^ The deposition pulse consisted of −1.0 V_SCE_ for 10 ms followed by −0.1 V_SCE_ for 20 ms. The XPS results showed partially oxidized Ni and reduced phosphorus, indicating that electrons were transferred from Ni to P. The optimized charge density of Ni and P was observed at the composition of Ni_78_P_22_, showing the highest HER activity with proton‐attracting ability. The Ni_78_P_22_/CP was used as the cathode for a PEMWE single cell, which demonstrated a cell voltage of 2.2 V at a current density of 3 A cm^−2^. Similarly, pulse electrodeposition was used to fabricate Co−P^[^ ^108^ ^]^ and Fe−P catalysts,^[^ ^104^ ^]^ showing high HER performance owing to the modified electronic structure.

Selective leaching is an effective post‐treatment for controlling the composition and morphology of transition metal phosphides. Nanoporous NiP was prepared on CP using electrochemical methods, including electrodeposition and subsequent selective leaching.^[^ ^109^ ^]^ The bulk Ni/P ratio of the catalyst was optimized by modulating the Ni precursor concentration in the electrodeposition bath. Then, electrochemical leaching was conducted on the as‐prepared NiP in 0.5 M H_2_SO_4_ at a constant potential. During the process, excess Ni was dissolved, resulting in a decrease in the Ni/P ratio of the catalyst and yielding a nanoporous structure. The optimized electrode showed an HER overpotential of 103 mV at −10 mA cm^−2^ in a half‐cell test and a current density of 1.47 A cm^−2^ at a 2.0 V_cell_ in a single‐cell test.

The fabrication of binary metal phosphides further enhanced HER activity. Kim et al. prepared Fe‐based binary metal phosphide catalysts on CP using an electrodeposition method.^[^ ^106^ ^]^ All Fe based metal phosphide catalyst demonstrated spherical shapes. Among these, FeCoP showed the highest intrinsic HER activity. The catalyst was electrodeposited on CF/CP to increase the ECSA. The FeCoP/CF/CP exhibited an overpotential of 125 mV at −10 mA cm^−2^ and a Tafel slope of 136 mV dec^−1^ in 0.5 M H_2_SO_4_ electrolyte. Furthermore, a PEMWE single‐cell with a FeCoP/CF/CP cathode demonstrated a current density of 0.95 A cm^−2^ at 2.0 V_cell_. Similarly, Cao et al. fabricated a pine‐needle‐like dendrite nanotube Ni‐Cu‐P catalyst on a CP with a larger surface using an in‐situ electrodeposition method.^[^ ^105^ ^]^ By changing the concentration of the Cu precursor, Ni atoms were partially replaced with Cu, thereby modifying the electronic structure. The pine‐needle‐like dendrite nanotube structure can be obtained with appropriate Cu precursor concentration. The optimized electrode showed an overpotential of 150 mV at −10 mA cm^−2^ and a Tafel slope of 69 mV dec^−1^ in 0.5 M H_2_SO_4_ electrolyte.

Kim et al. reported an amorphous ternary Co‐P‐B catalyst on CP prepared by one‐step electrodeposition.^[^ ^107^ ^]^ Varying the P and B precursor concentrations in the electrolyte controlled the atomic composition. The Co‐P‐B/CP electrode showed a spherical shape with a size of 100–200 nm, and its amorphous structure was confirmed by XRD and TEM analyses. The intrinsic HER activity of Co‐P‐B/CP was determined by normalizing the geometric HER activity by the ECSA. It was revealed that the intrinsic HER activity was significantly affected by the B/P ratio. The highest HER intrinsic activity was obtained when the B/P ratio was approximately 1, where electron transfer among the Co, P, and B elements was maximized.

Electrodeposited transition metal sulfides also exhibit high HER performance in acidic electrolytes. MoS*_x_* was electrodeposited on CP by varying the deposition parameters.^[^ ^110^ ^]^ The optimal electrode prepared at −1.0 V_SCE_ for 300 s showed an HER overpotential of 159.8 mV at −10 mA cm^−2^ and a Tafel slope of 42.3 mV dec^−1^ in 0.5 M H_2_SO_4_. XPS analysis indicated that the ratio of S atoms with higher binding energies affected the intrinsic HER activity. The prepared MoS*_x_*/CP electrodes were applied to a PEMWE single cell, which demonstrated a reasonable current density of 0.37 A cm^−2^ at 1.9 V_cell_. Murthy et al. fabricated Ni_1−_ *_x_* Mo*_x_* S on FTO substrates through electrodeposition.^[^ ^111^ ^]^ For the deposition, cyclic voltammetry (CV) was performed 25 cycles between −1.0 and 0.4 V_Ag/AgCl_ at a scan rate of 0.005 V s^−1^. As a composition‐optimized electrode, Ni_0.96_Mo_0.04_S exhibited an HER overpotential of 180 mV at −10 mA cm^−2^ and a Tafel slope of 50 mV dec^−1^ in 0.5 M H_2_SO_4_ (Figure 3F). The high activity originated from the faster discharge of protons in the initial Volmer step. Furthermore, long‐term electrolysis confirmed that 13% of the initial activity was lost during stability test at −0.2 V_RHE_ for 24 h. Meanwhile, the high HER activity of binary transition metal oxysulfides was reported by Kim et al.^[^ ^113^ ^]^ Using NiCo oxide electrodeposition and a subsequent S ion exchange reaction (IER) under ambient conditions, Ni*_y_* Co_1‐_ *_y_* O*_x_* S*_z_* was prepared on CP. As a first step, fern‐like Ni*_y_* Co_1‐_ *_y_* O*_x_*/CP was fabricated by galvanostatic electrodeposition by varying the metal precursor concentration. Then, a room‐temperature sulfidation process was performed through S IER in 1 mM Na_2_S solution for various reaction times. With an increase in the S IER time, the HER overpotential gradually decreased until 20 min and then saturated. The intrinsic HER activity increased with the S atom having a higher binding energy ratio. The optimized Ni_0.64_Co_0.36_O*_x_* S_0.28_/CP demonstrated an HER overpotential of 172 mV at −10 mA cm^−2^ and a Tafel slope of 78 mV dec^−1^ in 0.5 M H_2_SO_4_ electrolyte. A PEMWE single cell with the Ni_0.64_Co_0.36_O*_x_* S_0.28_/CP cathode demonstrated a reasonable current density of 0.72 A cm^−2^ at 2.0 V_cell_.

Recently, a heterostructured catalyst consisting of metal and metal oxides was proposed to modulate the hydrogen binding energy.^[^ ^99^, ^100^ ^]^ Typically, negative and positive Δ*G*_H*_ values for metal and metal oxides, respectively, can complement each other to obtain the optimized value. Han et al. fabricated a micro‐nanoporous MoO_2_@CoMo heterostructure on CP using two‐step electrochemical methods.^[^ ^99^ ^]^ By controlling the electrodeposition conditions, microporous coral‐like CoMo was formed on the CP. Subsequent electrochemical anodic etching selectively removed the excess Co and oxidized the Mo. TEM and XPS analyses confirmed the formation of MoO_2_ on the CoMo surface. The intrinsic HER activity gradually increased with increasing Mo^4+^ ratio by the synergetic effect, indicating that the surface composition of MoO_2_ increased owing to the properly modified Δ*G*_H*_ of CoMo (Figure 3H). The optimized catalyst exhibited a low HER overpotential of 76 mV at −50 mA cm^−2^ (Figure 3I). In addition, a long‐term test at −10 and −50 mA cm^−2^ revealed that the Mo^4+^ ratio slightly decreased at the initial stage and then saturated owing to the formation of irreversible insoluble hydrogen molybdenum bronze (H*_x_* MoO_3_). Similarly, the MoO_3_‐MoO_2_@NiMo/CP electrode showed excellent HER activity.^[^ ^100^ ^]^ As a first step, the NiMo film was electrodeposited on the CP, after which an applied anodic potential enabled the formation of MoO_2_ and MoO_3_ at the surface. The optimized electrode showed the lowest HER overpotential of 27.9 mV at −10 mA cm^−2^ and a Tafel slope of 25.3 mV dec^−1^ in 0.5 M H_2_SO_4_. Long‐term test proved the excellent stability of the electrode with near 100% of H_2_ Faradaic efficiency (FE). The electrode also exhibited a high HER activity in neutral and alkaline electrolytes.

Pretreatment of the substrate for electrodeposition can control the material properties of electrodeposits and thus, affects the HER activity. Dong et al. employed a pretreated CP substrate for deposition via electrochemical oxidation.^[^ ^112^ ^]^ After fabricating MoS_2_/CP nanosheet through a hydrothermal reaction, electrodeposition was performed in a solution containing Co and Ni precursors to produce CoNi LDH/MoS_2_/CP (CNL/MoS_2_/CP). The in‐situ reduction process was then used to produce the CN/CoNi LDH/MoS_2_/CP electrode. After the reduction process, CN nanoparticles have covered the precursor surface. The electrode showed an HER overpotential of 172 mV at −10 mA cm^−2^ and a Tafel slope of 77 mV dec^−1^ in 0.5 M H_2_SO_4_. DFT calculations supported that the 3D hybridized catalyst promoted the water dissociation reaction kinetics. The electrode also demonstrated excellent stability after 1000 CV cycles. Li et al. fabricated aligned graphene nanosheets (VAGNs) on CC through microwave plasma‐enhanced chemical vapor deposition as a substrate for FeOOH electrodeposition.^[^ ^101^ ^]^ Then, a low HER overpotential of 53 mV at −10 mA cm^−2^ and a Tafel slope of 42 mV dec^−1^ in 0.5 M H_2_SO_4_ was achieved through low‐temperature phosphidation. The 3D structure of VAGNs/CC provided a sufficiently accessible catalytic site. The strong π−π interaction of VAGN directly connected to the Fe atom site enabled faster electron transfer. Reduced graphene oxide (rGO) is also an effective support for electrodeposited catalysts.^[^ ^102^ ^]^ Liu et al. reported a two‐step fabrication of Fe_2_P@rGO on a Ti plate. First, FeO*_x_* @rGO was prepared by an electrodeposition method by varying the ratio of GO to the Fe precursor. Then, a phosphorization process in a furnace with a P precursor allowed the formation of the Fe_2_P@rGO catalyst, which showed an HER overpotential of 101 mV at −10 mA cm^−2^ and a Tafel slope of 55.2 mV dec^−1^ in 0.5 M H_2_SO_4_. The superior activity of the catalyst comes from the vertical Fe_2_P‐sandwiched rGO hybrid structure. Owing to this structure, superior electrical conductivity and fast electrolyte access are possible, and more active sites can be exposed.

#### 2.2.2 In alkaline media

The HER activity of transition‐metal‐based electrodes prepared by electrodeposition has also been investigated in alkaline media by varying their morphological and compositional properties.^[^ ^114^, ^115^, ^116^, ^117^ ^]^ Multicomponent alloys and compounds exhibit a synergistic effect among the elements that promote electron transfer and, as a result, improves the HER activity.^[^ ^118^, ^119^, ^120^, ^121^, ^122^, ^123^, ^124^, ^125^, ^126^, ^127^, ^128^, ^129^ ^]^ Table 6 summarizes the deposition conditions and HER activity of state‐of‐the‐art transition metal compounds in alkaline media.^[^ ^104^, ^114^, ^115^, ^116^, ^117^, ^118^, ^119^, ^120^, ^121^, ^122^, ^123^, ^124^, ^125^, ^126^, ^127^, ^128^, ^129^, ^130^, ^131^, ^132^, ^133^, ^134^, ^135^, ^136^, ^137^, ^138^, ^139^, ^140^, ^141^, ^142^, ^143^, ^144^, ^145^ ^]^

**TABLE 6:** Summary of deposition conditions and HER performance of non‐noble‐metal‐based electrodes in alkaline media

		Deposition condition							Overpotential (mV)						
Catalyst	Loading mass	Electrolyte	Potential/current/time	Substrate	Temperature (℃)	Post‐treatment	Electrolyte	Tafel slope (mV dec^−1^)	η_10_	η_20_	η_50_	Exchange current density (mA cm^−2^)	Stability	Single‐cell test (Y/N)	Ref.
c‐Ni@a‐Ni(OH)_2_	–	1 M NiCl_2_·6H_2_O + 0.2 M thioacetamide	−1.2 V_Hg/HgO_ for 200 s	Cu foam	–	–	1.0 M KOH	44.8	57	85	113	–	−10 mA cm^−2^ for 27 h	N	^[^ ^114^ ^]^
Foam 3	18.21 mg_catal_	0.1 M NiCl·6H_2_O + 1 M NH_4_Cl	−1 A/cm^2^ for 150 s	Stainless steel	–	–	8.0 M KOH	160	190	240	–	6.31 x 10^−1^	−0.35 V_RHE_ for 4 h	N	^[^ ^115^ ^]^
CoNi‐OOH‐30(40)	–	1.5 mM C_4_H_6_CoO_4_·4H_2_O + 1.5 mM NiCl_2_·6H_2_O + 1.5 mM NH_4_Cl	−10 mA/cm^2^ for 30 min	Titanium sheet	–	Electrochemical oxidation	1.0 M KOH	67	210	250	275	–	−10 mA cm^−2^ for 60 h	N	^[^ ^118^ ^]^
NiCo foam	4.3 mg cm^−2^	80 g L^−1^ NiCl_2_·6H_2_O + 20 g L^−1^ CoCl_2_·6H_2_O + 30 g L^−1^ H_3_BO_3_ + 200 g L^−1^ NH_4_Cl	0.05 A cm^−2^ for 5 min	Ni/Cu	60	–	1.0 M KOH	69.8	86.7	113	150	–	−100 mA cm^−2^ for 10 h	N	^[^ ^119^ ^]^
Porous Ni‐Co alloy	–	80 g L^−1^ NiCl_2_·6H_2_O + 20 g L^−1^ CoCl_2_·6H_2_O + NH_4_Cl + H_3_BO_3_	−2.5 A cm^−2^ for 120 s	Cu substrate	25	–	1.0 M KOH	26	54	107	–	–	−100 mA cm^−2^ for 10 h	N	^[^ ^120^ ^]^
Fe_50_Ni_50_	_–_	13.901 g L^−1^ FeSO_4_·7H_2_O + 13.1425 g L^−1^ NiSO_4_·6H_2_O + 6.183 g L^−1^ H_3_BO_3_	−1.5 V_Ag/AgCl_ for 30 s	Au/Si	–	–	1.0 M NaOH	96	390	440	–	–	–	N	^[^ ^121^ ^]^
Ni‐Fe	–	1 M NiCl_2_·6H_2_O + 30 g L^−1^ FeCl_2_·4H_2_O + 1.2 M CaCl_2_·2H_2_O	−40 mA cm^−2^ for 10 min	Cu sheet	60	–	1.0 M KOH	114	124	157	208	–	−100 mA cm^−2^ for 10 h	N	^[^ ^122^ ^]^
GO@Ni‐Cu@NF	–	0.5 M NiSO_4_·6H_2_O + 0.01 M CuSO_4_·5H_2_O + 0.02 M (NH_4_)_2_SO_4_ + 1 M HCl + 1.5 M H_2_SO_4_	−2 A cm^−2^ for 4.8 min	Nickel foam	25	GO deposition	1.0 M KOH	57	70	109	162	–	−100 mA cm^−2^ for 10 h	N	^[^ ^123^ ^]^
Ni_4_Mo/MoO*_x_*	0.27 mg cm^−2^	76.6 g L^−1^ NiCl_2_·6H_2_O + 6.6 g L^−1^ Na_2_MoP_4_·2H_2_O + 56.6 g L^−1^ Na_3_C_6_H_5_O_7_·2H_2_O	−50 mA cm^−2^ for 300 s	Cu foam	–	–	1.0 M KOH	64	16	49	83	–	−100 mA cm^−2^ for 24 h	N	^[^ ^124^ ^]^
Ni‐Co‐Fe	–	238 g L^−1^ NiCl_2_·6H_2_O + 30 g L^−1^ CoCl_2_·6H_2_O + 30 g L^−1^ FeCl_2_·4H_2_O + 31 g L^−1^ H_3_BO_3_ + 200 g L^−1^ C_2_H_10_Cl_2_N_2_	1^st^ step: −20 mA cm^−2^ for 10 min 2^nd^ step: −50 mA cm^−2^ for 1 min	Cu substrate	60	–	1.0 M KOH	86	91	119	153	–	−100 mA cm^−2^ for 30,000 s	N	^[^ ^125^ ^]^
Ni‐Fe‐Sn60	–	1 g L^−1^ NiSO_4_·6H_2_O + 0.25 g L^−1^ FeSO_4_·7H_2_O + 0.05 g L^−1^ SnSO_4_ + 0.18 g L^−1^ H_3_BO_3_ + 3 g L^−1^ NaCl + 0.68 g L^−1^ C_6_H_9_Na_3_O_9_	−20 mA cm^−2^ for 60 min	Ni mesh	25	–	1.0 M KOH	70	43	63	95	–	−10, −20, −50 mA cm^−2^ for 12 h	N	^[^ ^126^ ^]^
Ni‐Mo‐Fe	–	0.1 M NiCl_2_·6H_2_O + 0.02 M Na_2_MoO_4_·2H_2_O + 0.006 M FeCl_2_·4H_2_O + 0.3 M C_5_H_5_Na_3_O_7_·2H_2_O	−60 mA cm^−2^ for 60 min	Cu substrate	RT	–	1.0 M KOH	63	65	89	130	–	−100 mA cm^−2^ for 10 h	N	^[^ ^127^ ^]^
Ni‐Se‐Mo	–	100 g L^−1^ NiSO_4_·6H_2_O + 20 g L^−1^ SeO_2_ + 100 g L^−1^ LiCl + 20 g L^−1^ Na_2_MoO_4_·2H_2_O	−0.6 V_SCE_ for 600 s	Nickel foam	–	–	1.0 M KOH	98.9	101	134	183	9.93 x 10^−1^	−10 mA cm^−2^ for 20 h	N	^[^ ^128^ ^]^
Ni‐Se‐Cu	1.9 mg cm^−2^	100 g L^−1^ NiSO_4_·6H_2_O + 20 g L^−1^ SeO_2_ + 0.5 g L^−1^ CuSO_4_·5H_2_O + 100 g L^−1^ Na_3_C_6_H_5_O_7_·2H_2_O + 100 g L^−1^ LiCl	−0.6 V_SCE_ for 600 s	Nickel foam	40	–	1.0 M KOH	117.5	136	168	240	8.9 x 10^−1^	−10 mA cm^−2^ for 12 h	N	^[^ ^129^ ^]^
Co‐P/Cu NWs/CF	–	25 mM CoCl_2_ + 0.5 M NaH_2_PO_2_ + 25 mM CH_3_COONa	−1.1 V_Ag/AgCl_ for 600 s	Cu NWs/CF	–	–	1.0 M KOH	62	–	–	117	–	−200 mA cm^−2^ for 50 h	N	^[^ ^130^ ^]^
FeP	2.6 mg cm^−2^	0.72 M FeSO_4_·7H_2_O + 0.72 M NaH_2_PO_2_ + 1 vol.% formic acid	−1.7 V_SCE_ for 120 s	Cu foil	–	Electrochemical oxidation	1.0 M KOH	60	110	–	–	^–^	−10 mA cm^−2^ for 24 h	N	^[^ ^104^ ^]^
FeP	–	25 mM FeSO_4_·7H_2_O + 0.5 M NaH_2_PO_2_·H2O	−0.9 V_SCE_ for 30 min	CP	–	Acidic immersion	1.0 M KOH	61.9	146	155	192	–	Potential step of 0.01 V per 500 s from −0.17 V_RHE_ to −0.22 V_RHE_	N	^[^ ^131^ ^]^
Co‐Ni‐P‐4	13 mg	0.04 M Ni(NO_3_)_2_ + 0.01 M Co(NO_3_)_2_ + 0.25 M C_2_H_3_NaO_2_ + 0.25 M NaH_2_PO_2_	CV: −0.3 ∼ −1.0 V_SCE_, 45 cycles, 5 mV s^−1^	Nickel foam	–	–	1.0 M KOH	87.6	96	110	–	–	−10 mA cm^−2^ for 10 h	N	^[^ ^132^ ^]^
Ni_0.1_Co_0.9_P	–	238 g L^−1^ NiCl_2_·6H_2_O + 31 g L^−1^ H_3_BO_3_ + 200 g L^−1^ C_2_H_10_C_12_N_2_	1^st^ step: −10 mA cm^−2^ for 10 min 2^nd^ step: −50 mA cm^−2^ for 1 min	Cu substrate	60	–	1.0 M KOH	153	51	74	96	–	−100 mA cm^−2^ for 50 h	N	^[^ ^133^ ^]^
Ni‐Mo‐P/NF	–	0.1 M NiCl_2_·6H_2_O + 0.02 M Na_2_MoO_4_·2H_2_O + 0.1 M NaH_2_PO_2_·H_2_O + 0.3 M C_6_H_5_Na_3_O_7_·2H_2_O	−60 mA cm^−2^ for 60 min	Nickel foam	–	–	1.0 M KOH	87.3	63	87	120	–	−100 mA cm^−2^ for 10 h	N	^[^ ^134^ ^]^
Cu‐Co‐P_20_ foam	0.76 mg cm^−2^	0.04 M CuSO_4_ + 0.4 M CoSO_4_ + 1 M NaCl + 1 M H_2_SO_4_ + 0.02 M NaH_2_PO_2_	−2 A cm^−2^ for 20 s	Stainless steel	–	–	1.0 M KOH	48	138	160	–	–	−10 mA cm^−2^ for 6 h	N	^[^ ^135^ ^]^
Ni/NiS	–	0.05 M NiCl_2_ + 0.1 M CS(NH_2_)_2_ + 0.15 M NH_4_F + 0.2 M NaH_2_PO_2_·H_2_O	1^st^ step: −0.5 mA cm^−2^ for 90 min 2^nd^ step: −10 mA cm^−2^ for 75 min	Nickel foam	–	–	1.0 M KOH	79.9	43.8	68	100	–	−10 mA cm^−2^ for 20 h	N	^[^ ^136^ ^]^
Ni‐S alloy	–	100 g L^−1^ NiSO_4_·6H_2_O + 20 g L^−1^ NaCl + 40 g L^−1^ H_3_BO_3_ + 100 g L^−1^ CH_4_N_2_S + 5 g L^−1^ C_7_H_6_O_6_S + 70 g L^−1^ Na_3_C_6_H_5_O_7_·2H_2_O	−0.9V_SCE_ for 20 min	Nickel foam	40	–	1.0 M KOH	81.6	58	77	111	–	−50 mA cm^−2^ for 10 h	N	^[^ ^137^ ^]^
NiCoS_0.14_O_3.25_ NSs/NF	–	10 mM CH_4_N_2_S + 100 mM CoCl_2_ + 50 mM NiCl_2_	−0.8 V_Ag/AgCl_ fpor 1 h	Nickel foam	–	–	1.0 M KOH	94.3	170	–	–	–	−0.17 V_RHE_ for 18 h	N	^[^ ^138^ ^]^
S‐NiCo@50	–	0.032 M CoSO_4_·7H_2_O + 0.118 M NiSO_4_·6H_2_O + 0.359 M H_3_BO_3_ + 0.38 M NaCl + 6.3 mM C_6_H_8_O_7_·H_2_O + 0.584 M thiourea	−50 mA cm^−2^ for 8 min	Nickel foam	30	–	1.0 M NaOH	83	28	53	78	4.8	−0.085 V_RHE_ for 24 h	N	^[^ ^139^ ^]^
Ni‐Co‐S/CP	–	10 mM CoSO_4_·7H_2_O + 10 mM NiCl_2_·6H_2_O + 70 mM H_3_BO_3_ + 51 mM Na_3_C_6_H_5_O_7_·2H_2_O + 200 mM CH_4_N_2_S + 86 mM NaCl	−2.0 V_Hg/HgO_ for 1800 s	Carbon paper	–	–	1.0 M KOH	75	91	116	139	–	−0.4 A cm^−2^ for 8 h	Y	^[^ ^140^ ^]^
NiSe/NF	–	35 mM SeO_2_ + 200 mM LiCl + 65 mM Ni(CH_3_COO)_2_	−0.8 V_Ag/AgCl_ for 25 min	Nickel foam	–	–	1.0 M KOH	76.6	–	–	176	–	−50 mA cm^−2^ for 12 h	N	^[^ ^141^ ^]^
NiSe/NF	–	65 mM NiCl_2_·6H_2_O + 35 mM SeO_2_ + 20 mM LiCl	Pulse: 0 ∼ −0.8 V_SCE_, 1800 cycles, 0.01 Hz	Nickel foam	–	–	1.0 M KOH	89	65	87	–	–	−50 mA cm^−2^ for 20 h	N	^[^ ^142^ ^]^
NiCoSe_2_	1.6 mg_catal_ cm^−2^	10 mM NiCl_2_·6H_2_O + 10 mM CoCl_2_·6H_2_O + 20 mM SeO_2_ + 100 mM LiCl	−0.8 V_SCE_ for 600 s	Carbon cloth	–	–	1.0 M KOH	65	112.7	148	192	–	−10 mA cm^−2^ for 24 h	N	^[^ ^143^ ^]^
CNSe‐3.0	1.6 mg_catal_ cm^−2^	10 mM Co(NO_3_)_2_·7H_2_O + 20 mM Ni(NO_3_)_2_·7H_2_O + 40 mM Na_2_SeO_3_ + 100 mM LiCl	−0.8 V_Ag/AgCl_ for 3 min	Nickel foam	–	–	1.0 M KOH	76	184	220	313	–	−10 mA cm^−2^ for 20 h	N	^[^ ^144^ ^]^
Ni‐Fe‐Se/NF	6.86 mg_catal_ cm^−2^	1.2 mM SeO_2_ + 0.8 mM Ni(NO_3_)_2_·7H_2_O + 1.6 mM Fe(NO_3_)_3_	−1.5 V_Ag/AgCl_ for 10 min	Nickel foam	–	–	1.0 M KOH	102	85	108	156	–	−10 mA cm^−2^ for 50 h	N	^[^ ^145^ ^]^

Bubble‐templating electrodeposition is an effective strategy for porous catalyst fabrication having numerous active sites.^[^ ^115^, ^119^, ^120^ ^]^ Siwek et al. prepared a 3D Ni foam electrode using bubble‐templating electrodeposition with various concentrations of NH_4_Cl and NaCl in a deposition electrolyte.^[^ ^115^ ^]^ The electrolyte composition was found to affect the porosity and morphology of the 3D Ni foam. The HER activity of the optimized Ni foam exhibited an overpotential of 190 mV at −10 mA cm^−2^ in 8.0 M KOH electrolyte. The fabrication strategy can also be used to prepare bimetallic foam structures.^[^ ^120^ ^]^ Porous Ni‐Co foam electrodes were fabricated by modulating the applied current density and time. The pore diameter increased with increasing deposition time. The optimized electrode showed an HER overpotential of 54 mV at −10 mA cm^−2^ and a Tafel slope of 26 mV dec^−1^ in a 1.0 M KOH electrolyte. In the foam structure, the formation of a catalytic overlayer takes advantage of an enlarged ECSA. Nanostructured Ni‐Co alloys on 3D porous Ni foams were fabricated using two‐step electrodeposition.^[^ ^119^ ^]^ First, the 3D porous Ni foam was electrodeposited on a Cu substrate utilizing a galvanostatic method via a hydrogen bubble templating strategy. Subsequently, Ni‐Co alloy was electrodeposited on the as‐prepared 3D porous Ni foam, controlling the deposition current density to obtain different morphologies such as nanocones, nanoleaves, and nanoflakes. Among them, the Ni‐Co nanocones exhibited superior HER performance with an overpotential of 86.7 mV at −10 mA cm^−2^ and a Tafel slope of 69.8 mV dec^−1^. In addition, the Ni‐Fe‐Co nanocones electrodeposited on the Cu substrate showed a low HER overpotential of 91 mV at −10 mA cm^−2^ and a Tafel slope of 86 mV dec^−1^ owing to the facile H_2_ bubble release, elemental synergy, and lower charge transfer resistance.^[^ ^125^ ^]^

**FIGURE 4:**
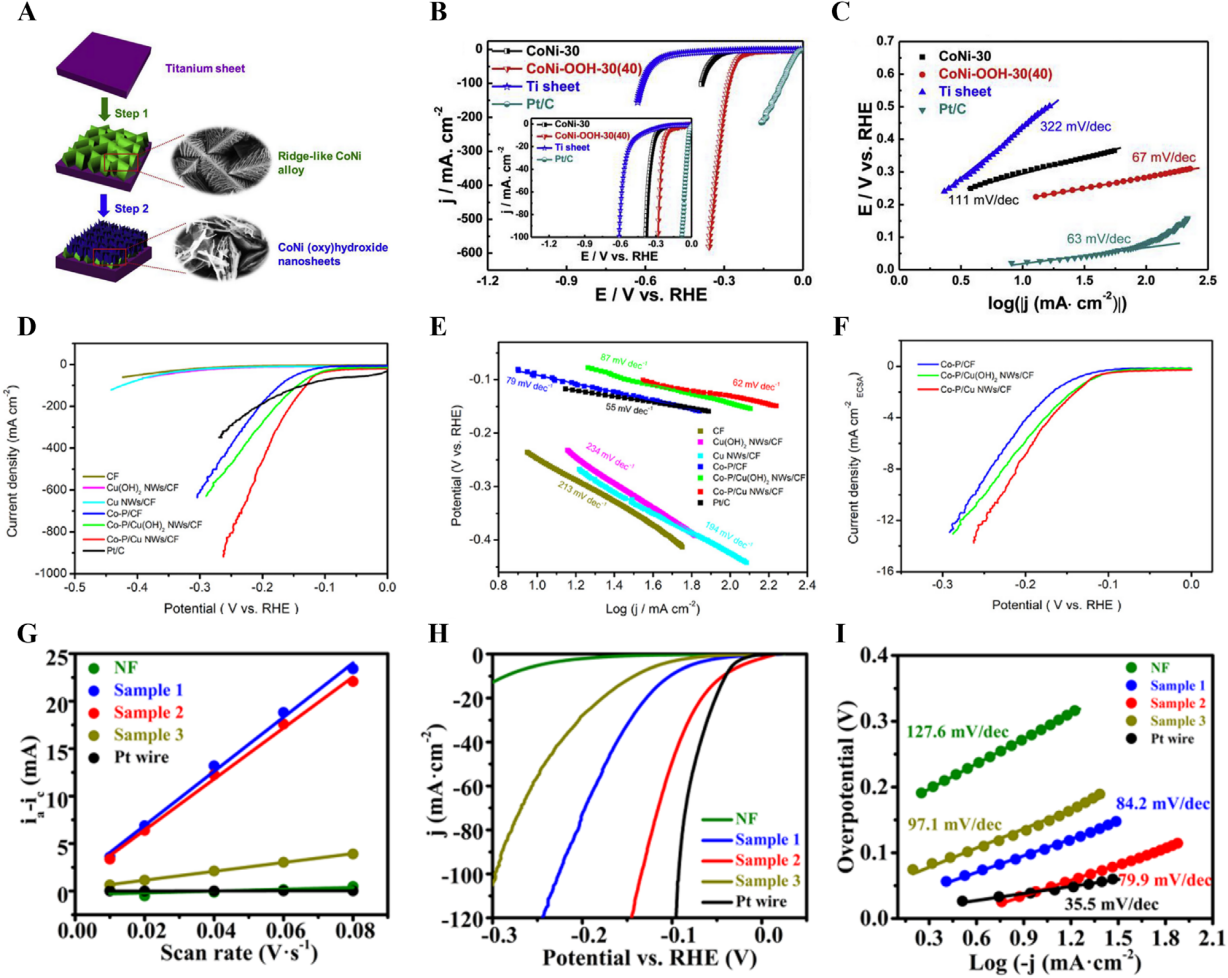
Self‐supported non noble metal‐based electrodes for HER in alkaline medium. (A) Schematic image of the synthesis process of CoNi oxyhydroxide catalyst. (B,C) Polarization curves and Tafel plots of as‐prepared CoNi oxyhydroxide catalyst in 1.0 M KOH. Reproduced with permission.^[^ ^118^ ^]^ Copyright 2019, Elsevier. (D,E) Polarization curves and Tafel plots of as‐prepared electrocatalyst in 1.0 M KOH. (F) HER activity normalized by ECSA. Reproduced with permission.^[^ ^130^ ^]^ Copyright 2021, Elsevier. (G) Double layer capacitance of as‐prepared amorphous Ni/NiS catalysts. (H,I) Polarization curves and Tafel plots of as‐prepared Ni/NiS catalysts in 1.0 M KOH. Reproduced with permission.^[^ ^136^ ^]^ Copyright 2021, American Chemical Society

The combination of metal and metal hydroxide showed a synergetic effect on the improvement of HER activity. For example, Hu et al. reported a crystalline–amorphous Ni‐Ni(OH)_2_ core–shell catalyst fabricated by one‐step thioacetamide (TAA)‐assisted electrodeposition.^[^ ^114^ ^]^ The TAA concentration in the deposition electrolyte played a crucial role in the formation of amorphous Ni(OH)_2_ nanosheets. The Ni‐Ni(OH)_2_ core–shell catalyst demonstrated an HER overpotential of 57 mV at −10 mA cm^−2^ and a Tafel slope of 44.8 mV dec^−1^. In addition, the HER performance at −10 mA cm^−2^ was stable during 27 h. DFT calculations suggested that the amorphous Ni(OH)_2_ shell showed a high H* adsorption ability, whereas the crystalline Ni core enhanced electron distribution for the Ni(OH)_2_ shell. The two‐step electrochemical process enabled the fabrication of a CoNi oxyhydroxide electrode.^[^ ^118^ ^]^ First, electrodeposition was conducted to prepare ridge‐like CoNi on a Ti sheet, and electrochemical oxidation was then employed to form oxyhydroxide (Figure 4A). After 40 h of electrochemical oxidation, the CoNi oxyhydroxide electrode showed an HER overpotential of 210 mV at −10 mA cm^−2^ and a Tafel slope of 67 mV dec^−1^, which are much higher than those of pristine CoNi (Figure 4B,C). The ultrathin nanosheet with a ridge‐like morphology provided a number of active sites and reaction interfaces, while also facilitating H_2_ bubble desorption on the catalyst surface.

Over the past few decades, many research groups have focused on the development of transition metal compounds such as phosphides,^[^ ^104^, ^130^, ^131^ ^]^ sulfides,^[^ ^136^, ^137^, ^138^, ^139^, ^140^ ^]^ and selenides.^[^ ^141^, ^142^, ^143^, ^144^, ^145^ ^]^ Among them, transition metal phosphides have received much attention because of their high catalytic activity for the HER, resulting from the modified electronic structure. For example, Yue et al. electrodeposited CoP nanosheets on Cu NW/CF prepared by a chemical oxidation process.^[^ ^130^ ^]^ The CoP/Cu NWs/CF electrode exhibited enhanced HER catalytic activity with an overpotential of 117 mV at −50 mA cm^−2^ and a Tafel slope of 62 mV dec^−1^ owing to the synergistic effect between the highly active Co‐P and highly conductive Cu NWs (Figure 4D,E). Furthermore, the stability test demonstrated that the HER performance at −200 mA cm^−2^ was stable for 50 h. Lu et al. reported the development of an Fe‐P electrode fabricated by two‐step electrochemical methods.^[^ ^104^ ^]^ A phosphide‐rich Fe‐P film was prepared by Fe‐P electrodeposition followed by electrochemical etching. The modified electronic structure showed a positive effect on HER activity with an overpotential of 110 mV at −10 mA cm^−2^ and a Tafel slope of 60 mV dec^−1^ in a 1.0 M KOH electrolyte. Similarly, high HER activity of nanoporous Fe‐P cubes was reported by Shi et al.^[^ ^131^ ^]^ They explained that the synergistic effects of the large active sites of 3D nanoporous structures, enhanced electronic conductivity of the pure Fe‐P phase, and binder‐free structures contributed to the high performance of HER in alkaline media. Bimetallic phosphides have also demonstrated high HER activity.^[^ ^133^, ^134^, ^135^ ^]^ For example, electrodeposition with CV allowed the formation of an amorphous Co‐Ni‐P catalyst on Ni foam.^[^ ^132^ ^]^ The number of cycles was varied to control the amount of deposited Co‐Ni‐P film. Its excellent HER performance mainly originates from the electronic interaction between binary metals and the large ECSA of the unique morphology.

Transition metal sulfides are also in the spotlight owing to their high HER activity and electrical conductivity. Wu et al. prepared the Ni‐S alloy by potentiostatic electrodeposition on Ni foam, which showed a high HER activity with an overpotential of 58 mV at −10 mA cm^−2^ and a Tafel slope of 81.6 mV dec^−1^.^[^ ^137^ ^]^ Similarly, electrodeposition was used to fabricate an amorphous Ni/NiS film by varying the reductant reagent to modulate the Ni content (Figure 4G–I).^[^ ^136^ ^]^ By controlling the composition, electronic structure, charge transfer resistance, and ECSA of the Ni/NiS film were optimized, which contributed to the high HER performance. Among them, the Ni/NiS film with a Ni/S ratio of 3.09 exhibited an HER overpotential of 43.8 mV at −10 mA cm^−2^ and a Tafel slope of 79.9 mV dec^−1^ (Figure 4H,I). Bimetallic sulfides also exhibited high HER activity with good durability.^[^ ^140^ ^]^ The morphologies and compositions of the Ni‐Co‐S catalyst electrodeposited on porous CP were controlled by varying the deposition parameters. The optimized Ni‐Co‐S/CP electrode with a S ratio of 8.97 demonstrated high HER activity with an overpotential of 91 mV at −10 mA cm^−2^. Furthermore, the optimized electrode was directly used as a cathode for an AEMWE, which exhibited a current density of 1.7 A cm^−2^ at 2.4 V_cell_. Meanwhile, the high HER activity of S‐doped NiCo film on Ni foam has been reported with an overpotential of 28 mV at −10 mA cm^−2^ in a 1.0 M NaOH electrolyte.^[^ ^139^ ^]^ Metallic Ni and Co efficiently enhanced the charge transport, while the synergistic effects between amorphous Ni*_x_* Co*_y_* S_(_ *_x_*_+_ *_y_*_)_ and crystalline metals contributed to improving the HER activity. Similarly, Li et al. reported the high HER activity of NiCoS*_x_* O*_y_* NSs on a Ni foam electrode fabricated by one‐step electrodeposition.^[^ ^138^ ^]^ The 3D structure of the Ni foam provided high electrical conductivity and a large ECSA as a metallic substrate. The electrode exhibited superior HER performance with an overpotential of 170 mV at −10 mA cm^−2^ and a low Tafel slope of 94.3 mV dec^−1^. In addition, the long‐term stability test was conducted at −0.17 V_RHE_ for 18 h showing stable performance. The synergistic effect with S ligand modulation, large active sites, self‐assembled nanosheet structures, and enhanced charge transport without binders lead to superior catalytic HER performance in alkaline media.

Electrodeposited transition metal selenides exhibit unique catalytic activity for HER. Gao et al. reported electrodeposited NiSe on a Ni foam electrode for HER in an alkaline medium.^[^ ^141^ ^]^ The NiSe catalyst fully covered the Ni foam and provided numerous surface catalytic sites. The interconnected structure of the NiSe catalyst facilitated charge transfer, resulting in enhanced HER performance with an overpotential of 176 mV at −50 mA cm^−2^. Pulsed potential electrodeposition was also effective in fabricating NiSe catalysts on Ni foam.^[^ ^142^ ^]^ The pulse frequency was controlled to obtain a smooth surface morphology with cracks, which showed a higher HER performance with good stability than that of the electrode fabricated at a constant potential. The superior HER activity was attributed to the 3D structure with a large active area and binder‐free high adhesion of NiSe on the Ni foam substrate. For further improvement, the compositional effect of the bimetallic selenide electrode was investigated.^[^ ^143^, ^144^, ^145^ ^]^

### 2.3 Noble‐metal‐based electrodes for ECR

#### 2.3.1 CO product

**FIGURE 5:**
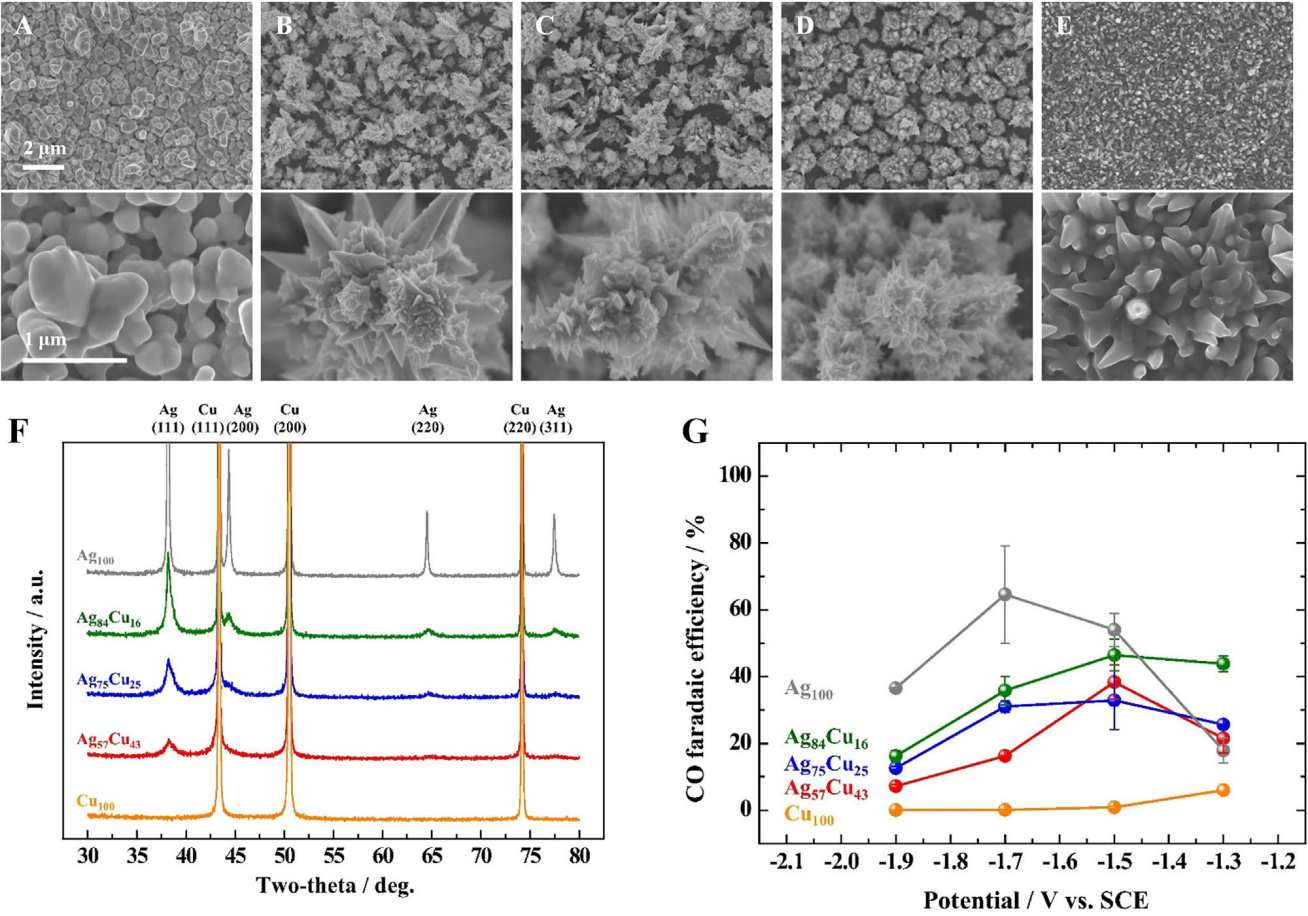
Self‐supported noble metal‐based electrodes for ECR to CO production. FESEM images of electrodeposited (A) Ag_100_, (B) Ag_84_Cu_16_, (C) Ag_75_Cu_25_, (D) Ag_57_Cu_43_, and (E) Cu_100_ (top line). Bottom line shows their expanded images. (F) XRD patterns of electrodeposited Ag_100_, Ag_84_Cu_16_, Ag_75_Cu_25_, Ag_57_Cu_43_, and Cu_100_. (G) CO Faradaic efficiencies of Ag, Ag‐Cu, and Cu dendrite catalyst. Reproduced with permission.^[^ ^150^ ^]^ Copyright 2016, Elsevier

**FIGURE 6:**
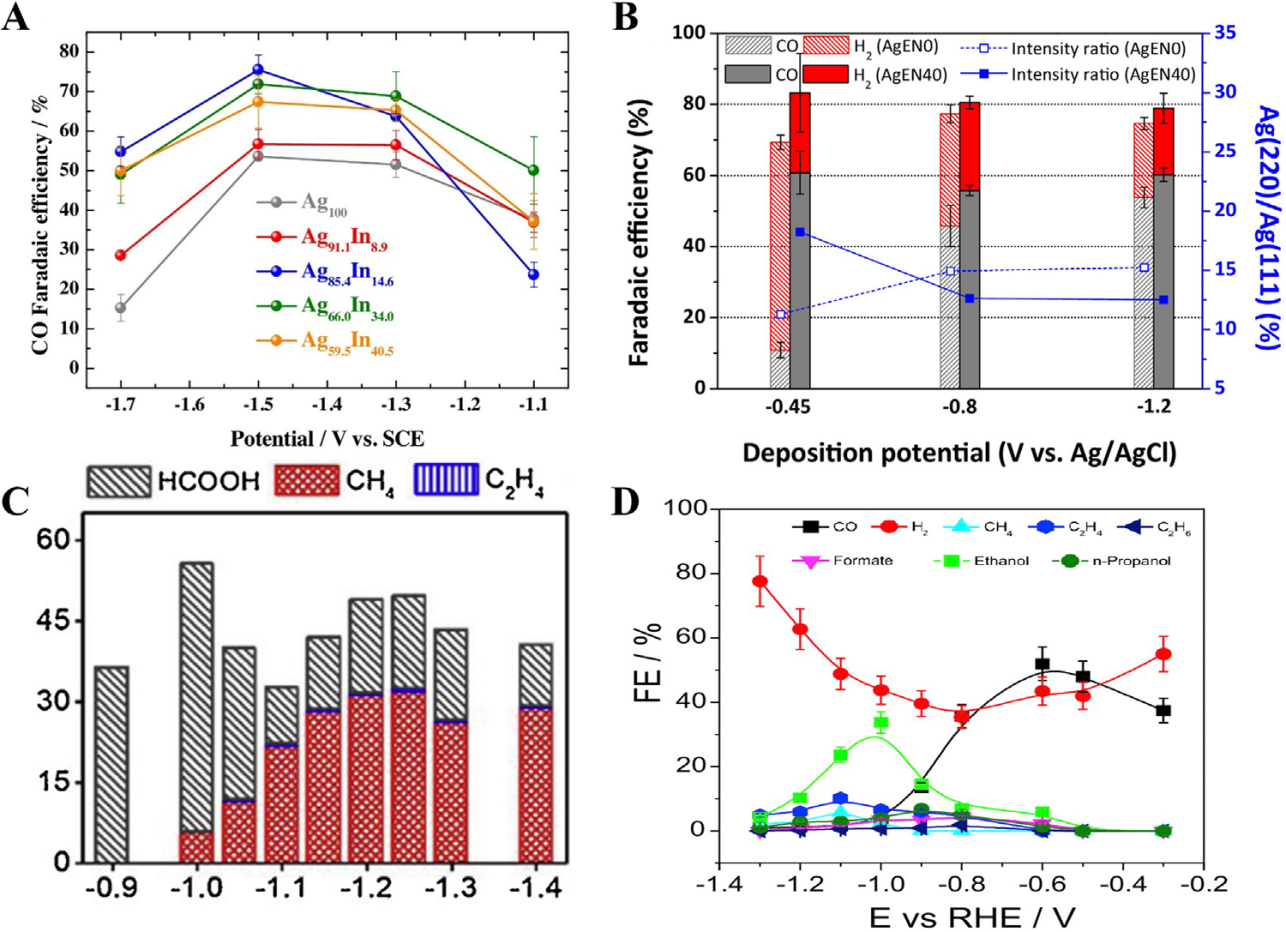
Self‐supported noble metal‐based electrodes for ECR. (A) CO Faradaic efficiency of Ag and AgIn dendrite catalysts. Reproduced with permission.^[^ ^149^ ^]^ Copyright 2017, Elsevier. (B) Comparison of CO Faradaic efficiency at −1.5 V with the Ag(220)/(111) intensity ratio of Ag catalysts prepared by controlling. Reproduced with permission.^[^ ^147^ ^]^ Copyright 2017, Elsevier. (C) CH_4_ Faradic efficiency of Cu‐Pd catalysts. Reproduced with permission.^[^ ^154^ ^]^ Copyright 2020, Elsevier. (D) C_2_H_5_OH Faradaic efficiency of the OD‐Ag_15_Cu_85_ catalyst. Reproduced with permission.^[^ ^157^ ^]^ Copyright 2020, Elsevier

For CO production, the reaction mechanism of the ECR involves three sequential steps. First, the CO_2_ dissolved in the electrolyte is adsorbed to the surface of the catalyst.^[^ ^59^ ^]^ The adsorbed CO_2_ then receives electrons, and the C–O bond of *COOH is broken. Finally, C≡O is desorbed from the surface of the catalyst. Among the noble metals, Au and Ag are generally employed as catalysts owing to their suitable binding energy for *COOH adsorption.^[^ ^146^, ^147^ ^]^ Moreover, various strategies using electrodeposition have been developed for the fabrication of efficient Au and Ag catalysts to increase their activity and selectivity through nanostructuring or alloying with transition metals, such as Cu (Figure 5A–G) or In (Figure 6A).^[^ ^148^, ^149^, ^150^, ^151^ ^]^ Deposition conditions and catalytic performances of noble‐metal‐based electrodes are summarized in Table 7.^[^ ^146^, ^147^, ^148^, ^149^, ^150^, ^151^, ^152^, ^153^, ^154^, ^155^, ^156^, ^157^, ^158^ ^]^

**TABLE 7:** Summary of deposition conditions and ECR performance of noble‐metal‐based electrodes

		Deposition condition										Cell type		
Catalyst	Loading mass	Electrolyte	Potential/current/time	Substrate	Temperature (℃)	Post‐treatment	Electrolyte	Target product	Faradaic efficiency	Partial current density (mA cm^−2^)	Stability	H‐type	Flow cell	Ref.
Au needle	–	0.5 M HCl + 160 mM HAuCl_4_	−0.4 V_Ag/AgCl_ for 300 s	Carbon paper	–	–	0.5 M KHCO_3_	CO	95% at −0.35 V_RHE_	–	−0.35 V_RHE_ for 8 h	O	X	^[^ ^146^ ^]^
AgEN40	–	10 mM AgNO_3_ + 40 mM C_2_H_8_N_2_ + 0.6 M (NH_4_)_2_SO_4_	−0.45 V_Ag/AgCl_ (1 C/cm^2^)	Cu foil	–	–	0.5 M KHCO_3_	CO	60.81% at −1.5 V_SCE_	−1.9	–	O	X	^[^ ^147^ ^]^
Au_61.1_Cu_38.9_ foam	5.15 mg_Au_ cm^−2^	0.12 M CuSO_4_·5H_2_O + 0.5 M H_2_SO_4_ + 1.2 M (NH_4_)_2_SO_4_ + 0.4 mM benzotriazole	1^st^ step: −2.5 mA cm^−2^ for 60 s 2^nd^ step: −1.2 A cm^−2^ for 40 s	Ti foil	–	Au displacement	0.5 M KHCO_3_	CO	41.3% at −0.73 V_RHE_	–	–	O	X	^[^ ^148^ ^]^
Ag_66.0_In_34.0_ dendrite	–	5 mM Ag_2_SO_4_ + 10 mM In_2_(SO_4_)_3_ + 1.0 M H_2_SO_4_ + 0.05 M C_6_H_8_O_7_	−1.5 V_NHE_ for 180 s	Cu foil	–	–	0.5 M KHCO_3_	CO	68.8% at −1.3 V_SCE_	–	–	O	X	^[^ ^149^ ^]^
Ag_57_Cu_43_ hierarchical dendrite	–	0.4 M KCN + 0.2 M CuCN + 10 mM M KAg(CN)_2_	−1.3 V_Ag/AgCl_ (400 mC/cm^2^)	Cu foil	RT	–	0.5 M KHCO_3_	CO	30.5% at −1.5 V_SCE_	–	–	O	X	^[^ ^150^ ^]^
AgCu‐50	95.24 μg_Ag_ cm^−2^	50 mM AgNO_3_ + 0.2 M CuSO_4_ + 1.5 M H_2_SO_4_	−1 A cm^−2^ for 10 s	Cu foil	–	–	0.1 M KHCO_3_	CO	58.4% at −0.6 V_RHE_	−2.35	−0.6 V_RHE_ for 5 h	O	X	^[^ ^151^ ^]^
Pd‐CV/FTO	–	0.1 g Pd(Cl)_2_ + 0.06 g NH_4_Cl + 0.2 g 2‐aminopyrodine in 200 mL H_2_O	CV: −1.43 ∼ 1.07 VRHE, 5 cycles, 50 mV s^−1^	FTO	–	–	0.1 M KHCO_3_	HCOO^−^	55% at −0.4 V_RHE_	–	−0.2 V_RHE_ for 20 h −0.3 V_RHE_ for 10 h	O	X	^[^ ^152^ ^]^
SnO*_x_*/AgO*_x_*	–	0.05 M SnCl_2_ + 2 M NaOH	1^st^ step: LSV of −0.75 to −2.0 V_Ag/AgCl_, 100 mV/s 2^nd^ step: −2.0 V_Ag/AgCl_ for 1 min	Ag foil	–	–	0.1 M KHCO_3_	HCOO^−^	5% at −0.8 V_RHE_	–	−0.8 V_RHE_ for 20 h	O	X	^[^ ^153^ ^]^
Cu‐Pd‐c3	–	0.04 M Cu(NO_3_)_2_ + 0.1 M NaCl + 0.68 mM Na_2_PdCl_4_	150 pulse cycles of + 0.02 V_Ag/AgCl_ 0.2 s and + 0.2 V_Ag/AgCl_ 0.02 s	Carbon paper	–	Electrochemical reduction	0.1 M KHCO_3_	CH_4_	32% at −1.25 V_RHE_	–	−1.2 V_RHE_ for 270 min	O	X	^[^ ^154^ ^]^
Ag foam	–	1.5 M H_2_SO_4_ + 0.02 M Ag_2_SO_4_ + 0.1 M Na_3_C_6_H_5_O_7_·2H_2_O	−3 A cm^−2^ for 20 s	Ag foil	–	–	0.5 M KHCO_3_	CH_4_	51% at −1.5 V_RHE_	–	−0.8 V_RHE_ for 72 h	O	X	^[^ ^155^ ^]^
CuAu NWA	–	0.02 M CuSO_4_ + 0.2 M glycine + 2 mM HAuCl_4_ + 10 mM ethylenediamine	3500 pulse cycles of −0.45 V_Ag/AgCl_ for 5 s, cell off and 1 mV_Ag/AgCl_ for 0.05 s	Au foil	25	–	0.1 M KHCO_3_	C_2_H_5_OH	45% at −0.7 V_RHE_	−1.0	−0.7 V_Ag/AgCl_ for 8 h	O	X	^[^ ^156^ ^]^
Ag_15_Cu_85_ foam	–	1.5 M H_2_SO_4_ + 20 mM CuSO_4_ + 2 mM Ag_2_SO_4_ + 0.1 M Na_3_C_6_H_5_O_7_·2H_2_O	−3.0 A/cm^2^ for 20 s	Cu foil	–	Annealing	0.5 M KHCO_3_	C_2_H_5_OH	33.7% at −1.0 V_RHE_	−8.6	−1.0 V_RHE_ for 100 h	O	X	^[^ ^157^ ^]^
AuCu/Cu‐SCA	–	30 mM CuSO_4_ + 2.5 mM NiSO_4_·6H_2_O + 240 mM NaH_2_PO_2_·H_2_O + 50 mM Na_3_C_6_H_5_O_7_·2H_2_O + 500 mM H_3_BO_3_ + 5 mg/L polyethylene glycol	−1.05 V_SCE_ for 30 min	Cu foil	65	Chemical reduction	0.5 M KHCO_3_	C_2_H_5_OH	29% at −1.0 V_RHE_	−5.5	−1.0 V_RHE_ for 24 h	O	X	^[^ ^158^ ^]^

Electrodeposition at a constant potential enabled the formation of a Au needle catalyst on a CP substrate.^[^ ^146^ ^]^ The Au needle catalyst exhibited a CO FE of 95% at −0.35 V_RHE_ in a 0.5 M KHCO_3_ electrolyte. The CO partial current density of the Au needle was 63 times higher than that of a Au rod and 122 times higher than that of Au particles. Furthermore, stability test at −0.35 V_RHE_ for 8 h has been demonstrated. The high CO selectivity originated from the sharp tip, generating a high local electric field, which concentrated the electrolyte cation. The two‐step electrochemical process increased the ECSA and reduced the Ag loading.^[^ ^148^ ^]^ On Ti foil, bubble‐templating Cu electrodeposition was conducted to fabricate CF, and the surface of the CF was then replaced by Au using galvanic displacement. The optimized Au_61.1_Cu_38.9_ foam has the Au loading mass of 5.15 mg_Au_ cm^−2^ and showed a CO FE of 41.3% at −0.73 V_RHE_ in 0.5 M KHCO_3_.

Ag electrodeposition was conducted on a pretreated Cu foil at a constant potential with a fixed charge density.^[^ ^147^ ^]^ In the deposition electrolyte, ethylenediamine (EN) was added to control the Ag(220)/Ag(111) ratio in the Ag deposit, which was proportional to the CO selectivity (Figure 6B). The optimized sample had dendritic morphology and showed a maximum CO FE of 60.8% at −1.5 V_SCE_ with a partial current density of −1.9 mA cm^−2^ in 0.5 M KHCO_3_. EN affected the chemical state of Ag to be reduced, thereby improving the efficiency of CO production. Ag‐based bimetallic electrodes have exhibited high CO selectivity. For example, AgIn dendrites were electrodeposited on pretreated Cu foil (Figure 6A).^[^ ^149^ ^]^ The Ag and In compositions were controlled by varying the deposition parameters. The maximum CO FE with an optimum Ag_66.0_In_34.0_ dendrite was 68.8% at −1.3 V_SCE_ in a 0.5 M KHCO_3_ solution because the presence of an appropriate In content suppressed the HER. Similarly, Choi et al. prepared Ag‐Cu dendrites by electrodeposition at a constant potential with a fixed charge density.^[^ ^150^ ^]^ Varying the precursor concentration in the deposition electrolyte controlled the composition of Ag‐Cu dendrites (Figure 5A–E). The electrodeposited Ag dendrite exists as polycrystalline Ag (Figure 5F). Among them, the Ag_57_Cu_43_ dendrite achieved the highest Ag mass‐normalized FE of 30.5% at −1.5 V_SCE_ in 0.5 M KHCO_3_, which was 2.2 times higher than that of Ag_100_ (Figure 5G). Meanwhile, bubble‐templating electrodeposition facilitated the fabrication of dendritic AgCu foam on Cu foil at a high current density for a short deposition time.^[^ ^151^ ^]^ The Ag loading mass of AgCu foam was 95.24 μg_Ag_ cm^−2^. The optimized AgCu foam electrode exhibited a maximum CO FE of 58.4% at −0.6 V_RHE_ with a partial current density of −2.4 mA cm^−2^ in 0.1 M KHCO_3_. The presence of Cu enhanced the Ag mass activity and long‐term stability of the pure Ag foam electrode.

#### 2.3.2 HCOO^–^ product

The ECR reaction mechanism to produce HCOO^–^ proceeds in three sequential steps.^[^ ^59^ ^]^ In the first step, CO_2_ dissolved in the electrolyte is adsorbed onto the surface of the catalyst in the form of *OCOH. Then, a proton–electron pair transfers to the *OCOH adsorbate, resulting in the production *OCOH_2_. Finally, HCOO^–^ is desorbed from the surface of the catalyst. Among the noble metals, Pd‐ and Ag‐based catalysts are generally used in the ECR to produce HCOO^–^. Furthermore, alloying with transition metals such as Sn or Bi reduces the overpotential with high selectivity.^[^ ^152^, ^153^ ^]^

A Pd nanostructure film was prepared on various substrates by two electrodeposition methods using CV and a constant potential.^[^ ^152^ ^]^ Among these, Pd‐CV on FTO showed the highest HCOO^–^ FE of 55% at −0.4 V_RHE_ in 0.1 M KHCO_3_. The Pd film grown by CV exhibited a more porous structure than those grown at constant potential. The Pd film fully covered by hydrogen suppressed CO production, which also improved the stability by avoiding CO poisoning on the Pd surface. Choi et al. fabricated SnO*_x_*/AgO*_x_* on O_2_‐plasma‐pretreated Ag foil by the electrodeposition of SnO*_x_*.^[^ ^153^ ^]^ At −0.8 V_RHE_ in 0.1 M KHCO_3_, the SnO*_x_*/AgO*_x_* electrode produced almost a 1:1 ratio of CO and HCOO^–^, whereas H_2_ was generated with a low FE of 5%. The presence of stable Sn^δ+^/Sn species stabilized the SnO*_x_*/AgO*_x_* surface under prolonged reaction times and enhanced the activity with a roughened surface. Stability test confirmed that it showed stable performance at −0.8 V_RHE_ for 20 h.

#### 2.3.3 CH_4_ product

CH_4_ production via the ECR requires eight proton–electron pairs. The reaction mechanism is similar to that of CO production, as previously described. Instead of desorption of C≡O, three proton–electron pairs transferring to the C≡O adsorbate result in the production of *CH_3_. Then, one more proton–electron pair is supplied to *CH_3_ to form CH_4_.^[^ ^159^ ^]^

Xie et al. fabricated a Cu‐Pd heterostructure catalyst on CP using pulsed cycle electrodeposition.^[^ ^154^ ^]^ The CH_4_ selectivity showed volcano behavior that was dependent on the Pd content. Tafel analysis suggested the promotion of reaction kinetics and enhancement of selectivity at the optimum Pd content. DFT calculations indicated that the hollow site of the Pd region in the Cu‐Pd heterostructure stabilized the CO* intermediate. Among them, the optimized Cu‐Pd heterostructure exhibited a superior CH_4_ FE of 32% at −1.25 V_RHE_ and a Tafel slope of 73.4 mV dec^−1^ in 0.1 M KHCO_3_ (Figure 6C). Additive‐assisted galvanostatic electrodeposition was conducted to fabricate Ag foam on Ag foil with micro‐ and macroporosity.^[^ ^155^ ^]^ The novel Ag foam catalyst demonstrated a unique feature—a remarkable CH_4_ FE of 51% at −1.5 V_RHE_—which corresponded to a partial current density of −18.8 mA cm^−2^. This superior Cu‐like behavior originated from the increased *CO binding energy of the Ag foam catalyst, which was higher than that of the polycrystalline Ag catalyst. However, long‐term tests showed severe degradation with morphological changes of the Ag foam catalyst in the CH_4_ forming region.

#### 2.3.4 C_2+_ product

In the reaction mechanism of the ECR, C_2+_ products require C‐C coupling of the reaction intermediates adsorbed on the catalyst surface. It is well known that Cu‐based catalysts enable the C‐C coupling pathway.^[^ ^60^ ^]^ In recent years, various studies have been conducted to control the oxidation number of Cu or fabricate alloys of Cu with other elements for the efficient production of C_2+_ chemicals.^[^ ^156^, ^157^, ^158^ ^]^

A single cycle of potentiostatic pulsed electrodeposition enabled the fabrication of a Cu*_x_* Au*_y_* nanowire array (NWA) with a high aspect ratio.^[^ ^156^ ^]^ Compared with the CuAu film, the CuAu NWA exhibited higher selectivity and lower onset potential, likely owing to the higher local pH and CO concentration near the NWA surface. The synergistic effect of morphology and surface electronic structure enhanced the adsorption of CO on the CuAu NWA; thus, they facilitated the subsequent reduction of *CO to C_2_H_5_OH through the C‐C coupling pathway. The optimized Cu*_x_* Au*_y_* NWA exhibited a maximum C_2_H_5_OH partial current density of −1 mA cm^−2^ at −0.7 V_RHE_ with an FE of 45% in 0.1 M KHCO_3_. Galvanostatic electrodeposition with bubble‐templating facilitated the preparation of AgCu foam on Cu foil, which was then annealed in air for further activation.^[^ ^157^ ^]^ The annealing procedure selectively oxidized the Cu to a mixture of crystalline Cu_2_O and amorphous CuO, whereas the Ag remained in the metallic state. Because the Cu oxide was reduced before ECR, the oxide‐derived Ag_15_Cu_85_ foam showed high selectivity for C_2_H_5_OH with facile transport of CO intermediates to Cu surface sites, which were particularly active for C‐C coupling. The oxide‐derived Ag_15_Cu_85_ foam exhibited a C_2_H_5_OH partial current density of −8.67 mA cm^−2^ at −1.0 V_RHE_ with an FE of 33.7% in 0.5 M KHCO_3_ (Figure 6D). It demonstrated a stability test at a potential of −1.0 V_RHE_ for 100 h. Shen et al. prepared a AuCu/Cu submicrocone array (SCA) with a two‐step route using electrodeposition, followed by an in‐situ chemical reduction method.^[^ ^158^ ^]^ The AuCu/Cu‐SCA showed a maximum C_2_H_5_OH FE of 29% at −1.0 V_RHE_ with a partial current density of −5.59 mA cm^−2^ in 0.5 M KHCO_3_. The stability of the catalyst was maintained for more than 24 h. It was revealed that the Au content had an impact on the partial current density of C_2_H_5_OH and C_2_H_4_ formation, as supported by DFT calculations showing modified binding energies of key intermediates.

### 2.4 Non‐noble‐metal‐based electrodes for ECR

#### 2.4.1 CO product

Electrodeposited Zn catalyst is mainly used to produce CO in the ECR.^[^ ^160^, ^161^, ^162^, ^163^, ^164^ ^]^ Deposition conditions and catalytic performance of non‐noble‐metal‐based electrodes are summarized in Table 8.^[^ ^160^, ^161^, ^162^, ^163^, ^164^, ^165^, ^166^, ^167^, ^168^, ^169^, ^170^, ^171^, ^172^ ^]^

**TABLE 8:** Summary of deposition conditions and ECR performance of noble‐metal‐based electrodes

		Deposition condition										
Catalyst	Loading mass	Electrolyte	Potential/current/time	Substrate	Temperature (℃)	Post‐treatment	Electrolyte	NH_3_ yield rate (μg h^−1^ mg^−1^_cat_)	Faradaic efficiency	Stability	Single‐cell test (Y/N)	Ref.
Porous Au film on Ni foam	–	30 mg PVP‐*co*‐PS + 3 mL THF + 1.5 mL alcohol + 3.5 mL HAuCl_4_ (40 mM)	−0.5 V_Ag/AgCl_ for 2000 s	Nickel foam	–	–	0.1 M Na_2_SO_4_	29.43 μg h^−1^ mg_Au_^−1^ at −0.2 V_RHE_	13.36% at −0.2 V_RHE_	−0.2 V_RHE_ for 20 h	N	^[^ ^175^ ^]^
AuPS alloy mesoporous film	–	10 mg PS‐*b*‐PEO+ 3 mL THF + 1.5 mL ethanol + 2 mL HAuCl_4_ (20 mM) + 20 mL NaH_2_PO_2_ (15 mM) + 1 mL Na_2_SO_3_ ( 10 mM)	−0.35 V_Ag/AgCl_ for 2000 s	Carbon paper	–	–	0.1 M Na_2_SO_4_	58.2 μg h^−1^ mg^−1^_cat_ at −0.2 V_RHE_	25.7% at −0.2 V_RHE_	−0.2 V_RHE_ for 20 h / 6 recycling test at −0.2 V_RHE_	N	^[^ ^176^ ^]^
Great stellated dodecahedral Au nanocrystals	–	Ageing ChCl–urea based DES solution containing 24.28 mM HAuCl_4_	1^st^ step: −0.97 V_Pt_ for 0.25 s 2^nd^ step: −0.50 V_Pt_ for 500 s	Glassy carbon disk	60	–	1 mM HCl	49.96 μg h^−1^ cm^−2^ at −0.4 V_RHE_	28.59% at −0.4 V_RHE_	6 recycling test at −0.4 V_RHE_	N	^[^ ^177^ ^]^
Excavated cubic Pt_93_Ir_7_	_–_	ChCl–urea based DES solution containing 0.74 mM IrCl_3_ and 9.26 mM K_2_PtCl_6_	CV: −0.20 ∼ −1.50 V_Pt quasi‐reference electrode_, 60 cycles, 50 mV s^−1^	Glassy carbon disk	80	–	1 mM HCl	28 μg h^−1^ cm^−2^ at −0.3 V_RHE_	40.8% at −0.3 V_RHE_	6 recycling test at −0.3 V_RHE_	N	^[^ ^178^ ^]^
Cu nano particle	–	0.5 M H_2_SO_4_ + 0.05 M CuSO_4_	−10 mA cm^−2^ for 2 min	Carbon paper	–	–	0.1 M KOH	12.0 μg h^−1^ mg^−1^_cat_ at 0.0 V_RHE_	33% in H‐type cell, 59% in Zn‐N_2_ battery at 0.0 V_RHE_	5 recycling test at −0 V_RHE_	N	^[^ ^179^ ^]^
Fe SAC/N−C	–	4 mg L^−1^ + Fe(NO_3_)_3_·9H_2_O + 1 g L^−1^ starch + 5 g L^−1^ H_3_BO_3_	−0.02 V_RHE_ with sonication for 3 h	Glassy carbon	30	Annealing	0.1 M KOH	53.12 μg h^−1^ mg^−1^_cat_ at −0.35 V_RHE_	39.6% at −0.35 V_RHE_	5 recycling test at −0.35 V_RHE_	N	^[^ ^180^ ^]^
Fe_2_O_3_/Cu	–	0.1 M CuCl_2_ + 0.1 M FeCl_2_	0.2, −0.7, 0.2 and −1.3 V_SHE_ each for 62.5 ms, 10 pulses	Ti sheet	–	–	0.1 M KOH	15.66 μg h^−1^ mg^−1^_cat_ at −0.1 V_RHE_	24.4% at −0.1 V_RHE_	−0 V_RHE_ for 12 h	N	^[^ ^181^ ^]^
Bi_2_O_3_/FEG	0.74 mg cm^−2^	0.3 g BiCl_3_ + 25 mL ethylene glycol	−1 mA cm^−2^ for 5 min	Functionalized exfoliated graphene	–	–	0.1 M Na_2_SO_4_	5.68 μg h^−1^ mg_Bi_^−1^	11.2% at −0.5 V_RHE_	−0.5 V_RHE_ for 12 h	N	^[^ ^182^ ^]^

**FIGURE 7:**
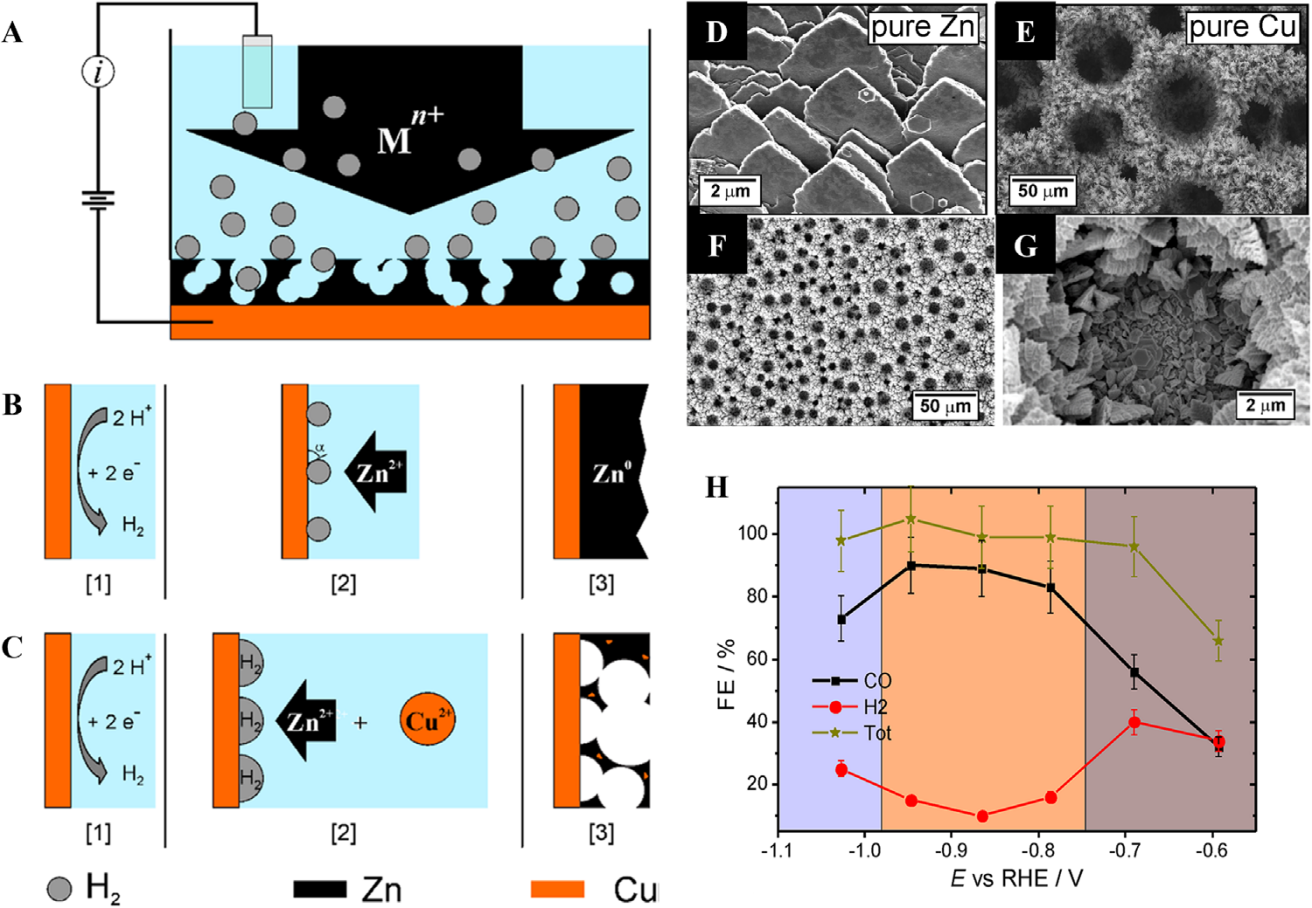
Self‐supported non noble metal‐based electrodes for ECR to CO production. (A) Sketch the principle of metal electrodeposition assisted by the DHBT method. (B) pure Zn fabrication by DHBT. (C) Zn‐Cu alloy fabrication by DHBT. SEM images of DHBT‐based electrodeposited (D) pure Zn, (E) pure Cu, and (F,G) Zn_94_Cu_6_ catalyst. (H) CO Faradaic efficiency of Zn_94_Cu_6_ catalyst. Reproduced with permission.^[^ ^163^ ^]^ Copyright 2018, American Chemical Society

**FIGURE 8:**
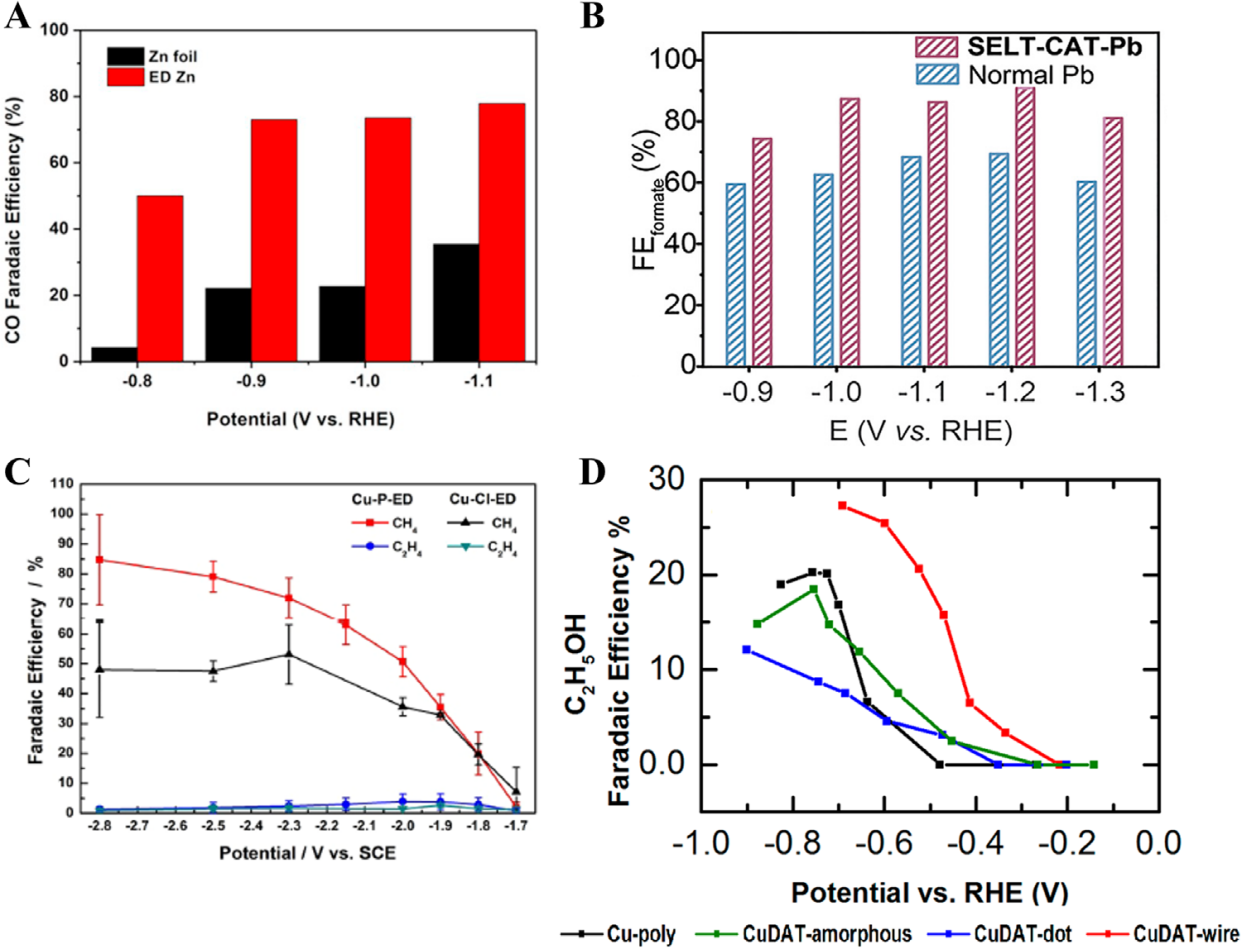
Self‐supported non noble metal‐based electrodes for ECR. (A) CO Faradaic efficiency of ED Zn catalyst and Zn foil. Reproduced with permission.^[^ ^161^ ^]^ Copyright 2018, Elsevier. (B) HCOOH Faradic efficiency of Pb‐based catalysts. Reproduced with permission.^[^ ^169^ ^]^ Copyright 2019, Elsevier. (C) CH_4_ Faradic efficiency of Cu‐based catalysts. Reproduced with permission.^[^ ^170^ ^]^ Copyright 2017, American Chemical Society. (D) C^2+^ products Faradaic efficiency of Cu‐based catalysts. Reproduced with permission.^[^ ^171^ ^]^ Copyright 2017, American Chemical Society

Rosen et al. prepared Zn dendrites on Zn foil surfaces by electrodeposition.^[^ ^160^ ^]^ The Zn dendrite electrode obtained using a deposition potential of −1.0 A cm^−2^ and a deposition time of 60 s showed an FE of 79% at −1.1 V_RHE_, with a partial current density of −13 mA cm^−2^ and a Tafel slope of 260 mV dec^−1^ in 0.5 M NaHCO_3_ in an H‐type cell. The Zn dendrite electrode exhibited a higher selectivity and activity than Zn foil by an order of magnitude. The oxidation state of the Zn dendrite was monitored by operando X‐ray absorption spectroscopy techniques and the oxidation number of the Zn dendrite was maintained at potentials above −0.7 V, explaining its excellent stability. Multistep electrodeposition also enabled the preparation of Zn dendrites on Zn foil,^[^ ^161^ ^]^ resulting in a unique nanoporous structure, which can provide large active sites. The ED Zn electrode showed a FE of 80% at −1.1 V_RHE_ in 0.5 M KHCO_3_ in a H‐type cell (Figure 7A). Luo et al. fabricated P‐Zn (porous Zn) on Cu mesh surfaces via electrodeposition.^[^ ^162^ ^]^ The P‐Zn electrode was obtained under a deposition time of 30 s and demonstrated an FE of 95% at −0.95 V_RHE_, with a partial current density of −26 mA cm^−2^ and a Tafel slope of 74 mV dec^−1^ in 0.1 M KHCO_3_ in an H‐type cell. Meanwhile, the P‐Zn electrode showed an FE of 84% at −0.64 V_RHE_, with a partial current density of −168 mA cm^−2^ in 1.0 M KHCO_3_ in a flow cell. The high catalytic performance was primarily attributed to the highly porous structure with numerous active sites and a local pH effect. Moreover, the binder‐free electrode prevented the blocking effect of the active sites. A Zn‐based bimetallic catalyst was fabricated via galvanostatic deposition (Figure 8A–G).^[^ ^163^ ^]^ At a constant current of −3 A cm^−2^ for 20 s, the obtained Zn_94_Cu_6_ catalyst achieved an FE of 90% at −0.95 V_RHE_, with a partial current density of 7.5 mA cm^−2^ in 0.5 M KHCO_3_ (Figure 8H). The stability of the catalyst was maintained for more than 36 h. An advanced approach utilized catalyst materials supported on a Cu mesh with open‐cell porosity, which enabled controlled flow through the catalyst framework of liquid flow. The Zn_94_Cu_6_ catalyst on Cu mesh exhibited an FE of 80% at −0.89 V_RHE_ in 0.5 M KHCO_3_ in a flow cell owing to the fast mass transport. Dendritic core‐shell Cu‐based materials were found to be suitable for CO production in the ECR.^[^ ^164^ ^]^ The electrodeposition method was employed to prepare In‐doped Cu@Cu_2_O catalyst on a CP substrate at a constant potential of −1.2 V_SCE_ for 500 s, which showed an FE of 87.6% at −0.8 V_RHE_ with a partial current density of –9.7 mA cm^−2^ in 0.1 M KHCO_3_. The Cu^+^/Cu^0^ ratio, ECSA, and catalytic performance could be adjusted by varying the amount of In dopant.

#### 2.4.2 HCOO^–^ product

Sn‐based materials are commonly used for the selective production of HCOO^–^ in the ECR.^[^ ^165^, ^166^, ^167^, ^168^ ^]^ Irtem et al. conducted galvanostatic Sn electrodeposition on a CP substrate to prepare GDEs for flow cell systems.^[^ ^165^ ^]^ The Sn GDE was fabricated at an applied constant current of −15 mA cm^−2^ for 5 min, showing uniform Sn coverage on the surface of the CP and 2.6 mg cm^−2^ of Sn loading mass. The Sn GDE reached an HCOO^–^ FE of 71% at −1.1 V_RHE_, with a partial current density of −8.3 mA cm^−2^ and a Tafel slope of 89 mV dec^−1^ in 0.5 M NaHCO_3_ in a flow cell. Moreover, this study demonstrated that FEs were also dependent on the gas/liquid flow ratio because the turbulence improved at the electrode in the flow cell. Wang et al. also fabricated a Sn‐based GDE via electrodeposition.^[^ ^166^ ^]^ The substrate consisted of carbon black, polytetrafluoroethylene, and copper mesh. Sn electrodeposition was conducted at a constant potential of −2.0 V_Ag/AgCl_ for 30, 60, 90, and 120 s. The optimized sample reached an HCOO^–^ FE of 73% at −1.1 V_RHE_, with a partial current density of −34 mA cm^−2^ in 0.5 M KHCO_3_ in a H‐type cell. Sn‐Cu bimetallic catalysts prepared by electrodeposition also exhibited high selectivity for HCOO^–^ production owing to the elemental synergy.^[^ ^167^, ^168^ ^]^ Wang et al. fabricated SELF‐CAT (self‐selective catalyst) on carbon paper by using self‐selective electrodeposition method.^[^ ^169^ ^]^ For fabrication of SELF‐CAT‐Pb and SELF‐CAT‐Cu, the deposition electrolyte was bubbled with CO_2_ for 20 min to reach saturation. The working electrode was applied at −0.66 V_RHE_ for a total of 4 C cm^−2^ with CO_2_ continuously bubbled in electrolyte. The SELF‐CAT‐Pb catalyst has octahedral nanoparticle structure and reaches a FE of 90.5% at −1.2 V_RHE_ with partial current density of −22 mA cm^−2^ in 0.5 M KHCO_3_ in a H‐type cell (Figure 7B), and then the SELF‐CAT‐Cu catalysts reach a FE of 54.5% at −0.7 V_RHE_.

#### 2.4.3 CH_4_ target product

Producing CH_4_ is less attractive than CO and HCOO^–^ owing to its lower price. Cu pulsed electrodeposition was performed on the CP substrate.^[^ ^170^ ^]^ The morphology of Cu was controlled by varying the ratio of deposition and etching currents. The optimized electrode with a roughened morphology achieved an FE of 85% at −2.8 V_RHE_, with a partial current density of −38 mA cm^−2^ and a Tafel slope of 88 mV dec^−1^ in 0.5 M NaHCO_3_ (Figure 7C). The surface morphology can significantly affect the product selectivity and catalytic activity for CH_4_ production.

#### 2.4.4 C_2+_ product

For ECR C_2+_ products, Cu‐based electrodes are commonly used to enhance the C‐C coupling reaction pathway. Hoang et al. electrodeposited Cu on a CP substrate in a deposition bath with or without additives.^[^ ^171^ ^]^ The Cu morphology changed significantly depending on the additives, pH, and applied current density. Among them, the Cu wires exhibited the C_2_H_4_ FE of 40% and C_2_H_5_OH FE of 20% at −0.5 V_RHE_ in 0.1 M KHCO_3_ (Figure 7D). Similarly, the dendritic morphology of Cu prepared at a constant potential was advantageous for the selective production of C_2+_.^[^ ^172^ ^]^ The optimized Cu showed a C_2_H_4_ FE of 32% at −1.7 V_Ag/Agcl_ with a partial current density of −4 mA cm^−2^ in 0.1 M KHCO_3_. By varying the morphology and crystal orientation, it was demonstrated that roughened Cu with highly populated (311) surfaces increased the selectivity of C_2_H_4_ production.

### 2.5 Noble‐metal‐based electrodes for NRR

Theoretical and experimental studies have suggested that Au, Ru, Rh, and Pd are efficient catalysts for NRR.^[^ ^173^, ^174^, ^175^, ^176^ ^]^ Au‐based catalysts are in the spotlight for high NRR selectivity owing to their less favorable hydrogen adsorption.^[^ ^175^, ^176^, ^177^ ^]^ Table 9 summarizes the NRR performance of noble‐ and non‐noble‐metal‐based electrodes fabricated by electrodeposition.^[^ ^175^, ^176^, ^177^, ^178^, ^179^, ^180^, ^181^, ^182^ ^]^

**TABLE 9:** Summary of deposition conditions and NRR performance of noble and non‐noble‐metal‐based electrodes

		Deposition condition										
Catalyst	Loading mass	Electrolyte	Potential/current/time	Substrate	Temperature (℃)	Post‐treatment	Electrolyte	NH_3_ yield rate (μg h^−1^ mg^−1^_cat_)	Faradaic efficiency	Stability	Single‐cell test (Y/N)	Ref.
Porous Au film on Ni foam	–	30 mg PVP‐*co*‐PS + 3 mL THF + 1.5 mL alcohol + 3.5 mL HAuCl_4_ (40 mM)	−0.5 V_Ag/AgCl_ for 2000 s	Nickel foam	–	–	0.1 M Na_2_SO_4_	29.43 μg h^−1^ mg_Au_^−1^ at −0.2 V_RHE_	13.36% at −0.2 V_RHE_	−0.2 V_RHE_ for 20 h	N	^[^ ^175^ ^]^
AuPS alloy mesoporous film	–	10 mg PS‐*b*‐PEO+ 3 mL THF + 1.5 mL ethanol + 2 mL HAuCl_4_ (20 mM) + 20 mL NaH_2_PO_2_ (15 mM) + 1 mL Na_2_SO_3_ ( 10 mM)	−0.35 V_Ag/AgCl_ for 2000 s	Carbon paper	–	–	0.1 M Na_2_SO_4_	58.2 μg h^−1^ mg^−1^_cat_ at −0.2 V_RHE_	25.7% at −0.2 V_RHE_	−0.2 V_RHE_ for 20 h / 6 recycling test at −0.2 V_RHE_	N	^[^ ^176^ ^]^
Great stellated dodecahedral Au nanocrystals	–	Ageing ChCl–urea based DES solution containing 24.28 mM HAuCl_4_	1^st^ step: −0.97 V_Pt_ for 0.25 s 2^nd^ step: −0.50 V_Pt_ for 500 s	Glassy carbon disk	60	–	1 mM HCl	49.96 μg h^−1^ cm^−2^ at −0.4 V_RHE_	28.59% at −0.4 V_RHE_	6 recycling test at −0.4 V_RHE_	N	^[^ ^177^ ^]^
Excavated cubic Pt_93_Ir_7_	_–_	ChCl–urea based DES solution containing 0.74 mM IrCl_3_ and 9.26 mM K_2_PtCl_6_	CV: −0.20 ∼ −1.50 V_Pt quasi‐reference electrode_, 60 cycles, 50 mV s^−1^	Glassy carbon disk	80	–	1 mM HCl	28 μg h^−1^ cm^−2^ at −0.3 V_RHE_	40.8% at −0.3 V_RHE_	6 recycling test at −0.3 V_RHE_	N	^[^ ^178^ ^]^
Cu nano particle	–	0.5 M H_2_SO_4_ + 0.05 M CuSO_4_	−10 mA cm^−2^ for 2 min	Carbon paper	–	–	0.1 M KOH	12.0 μg h^−1^ mg^−1^_cat_ at 0.0 V_RHE_	33% in H‐type cell, 59% in Zn‐N_2_ battery at 0.0 V_RHE_	5 recycling test at −0 V_RHE_	N	^[^ ^179^ ^]^
Fe SAC/N−C	–	4 mg L^−1^ + Fe(NO_3_)_3_·9H_2_O + 1 g L^−1^ starch + 5 g L^−1^ H_3_BO_3_	−0.02 V_RHE_ with sonication for 3 h	Glassy carbon	30	Annealing	0.1 M KOH	53.12 μg h^−1^ mg^−1^_cat_ at −0.35 V_RHE_	39.6% at −0.35 V_RHE_	5 recycling test at −0.35 V_RHE_	N	^[^ ^180^ ^]^
Fe_2_O_3_/Cu	–	0.1 M CuCl_2_ + 0.1 M FeCl_2_	0.2, −0.7, 0.2 and −1.3 V_SHE_ each for 62.5 ms, 10 pulses	Ti sheet	–	–	0.1 M KOH	15.66 μg h^−1^ mg^−1^_cat_ at −0.1 V_RHE_	24.4% at −0.1 V_RHE_	−0 V_RHE_ for 12 h	N	^[^ ^181^ ^]^
Bi_2_O_3_/FEG	0.74 mg cm^−2^	0.3 g BiCl_3_ + 25 mL ethylene glycol	−1 mA cm^−2^ for 5 min	Functionalized exfoliated graphene	–	–	0.1 M Na_2_SO_4_	5.68 μg h^−1^ mg_Bi_^−1^	11.2% at −0.5 V_RHE_	−0.5 V_RHE_ for 12 h	N	^[^ ^182^ ^]^

**FIGURE 9:**
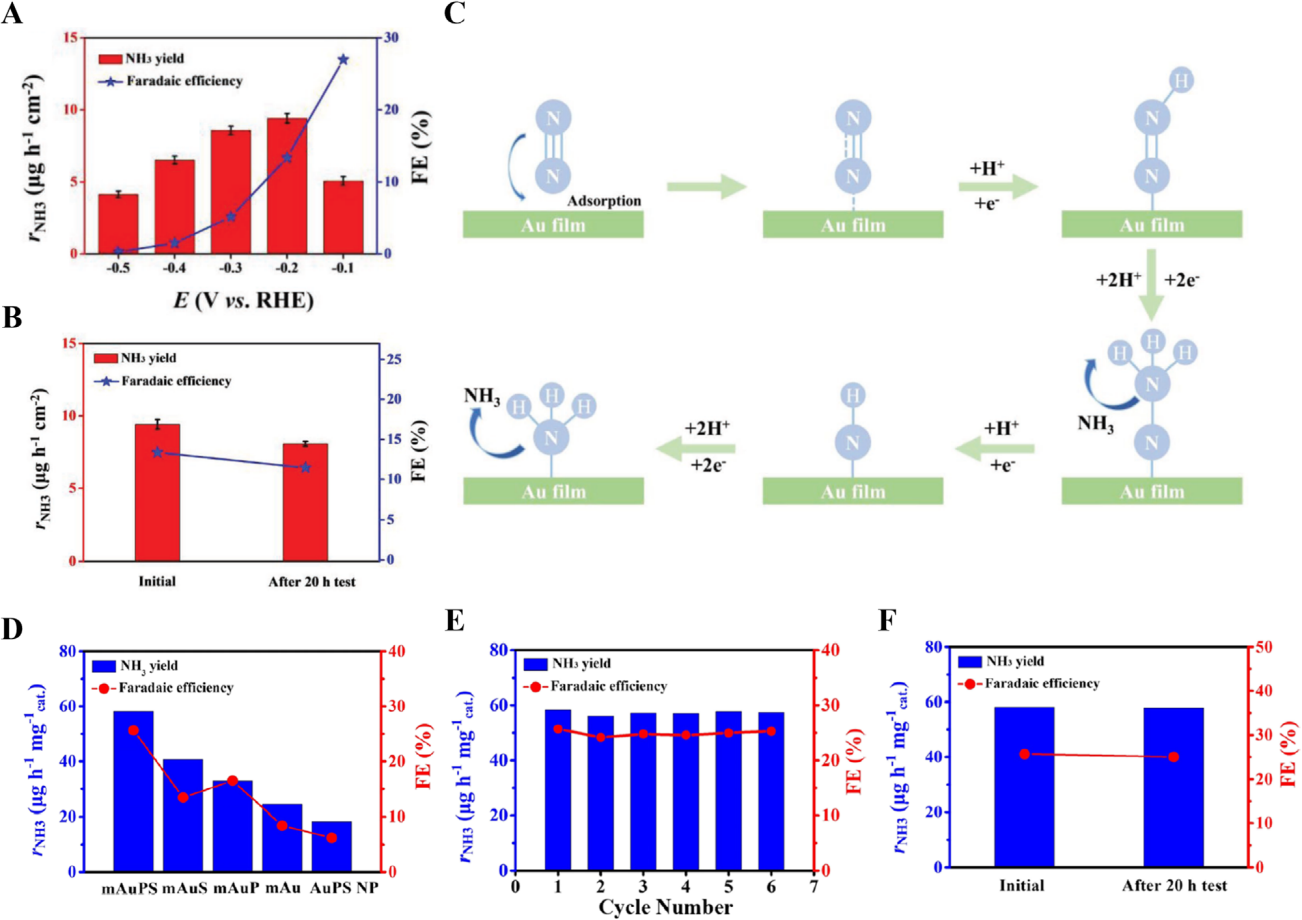
Self‐supported noble metal‐based electrodes for NRR. (A) NH_3_ yields and FEs of the pAu/NF at different potential in 0.1 M Na_2_SO_4_. (B) Comparison of NH_3_ yields and FEs for pAu/NF after long‐term stability test for 20 h. (C) Schematic illustration of the possible NRR pathway on the pAu/NF surface. Reproduced with permission.^[^ ^175^ ^]^ Copyright 2019, John Wiley and Sons. (D) NH_3_ yields and FEs of the Au‐based catalyst at −0.2 V_RHE_. (E) NH_3_ yields and FEs of the mAuPS/CP at −0.2 V_RHE_ with six recycling tests. (F) Comparison of NH_3_ yields and FEs for mAuPS/CP after long‐term stability test for 20 h. Reproduced with permission.^[^ ^176^ ^]^ Copyright 2021, American Chemical Society

Micelle‐assisted electrodeposition was conducted to fabricate Au film on Ni foam (pAu/NF).^[^ ^175^ ^]^ Using poly(1‐vinylpyrrolidone‐co‐styrene) as a soft template, an interconnected porous Au film with uniform nanopores could be prepared. The pAu/NF demonstrated a high NH_3_ yield rate of 29.4 μg h^−1^ mg_Au_^−1^ and an FE of 13.3% at −0.2 V_RHE_ in 0.1 M Na_2_SO_4_ (Figure 9A). In addition, the excellent stability of pAu/NF for NRR was confirmed; the current density of pAu/NF showed negligible degradation, and the NH_3_ yield rate decreased only slightly during the stability test at −0.2 V_RHE_ for 20 h (Figure 9B). The excellent NRR performance of the pAu/NF mainly originates from its spatially and locally nanoporous structures of Au active components. Furthermore, they propose a possible distal associative pathway for the NRR on the pAu/NF surface (Figure 9C). Dai et al. also employed micelle‐assisted electrodeposition to develop a P, S‐codoped mesoporous Au film on a CP substrate (mAuPS/CP).^[^ ^176^ ^]^ Compared to Au films with a single dopant, the mAuPS/CP showed higher NRR activity with an NH_3_ yield rate of 58.2 μg h^−1^ mg_cat_^−1^ and an FE of 25.7% at −0.2 V_RHE_ in 0.1 M Na_2_SO_4_ (Figure 9D). It was confirmed that the high NRR activity of mAuPS/CP originated from the strong adsorption of N_2_ intermediates by the d‐band center structure in the valence band. Moreover, the long‐term stability test for 20 h and the repetition of six consecutive tests at −0.2 V_RHE_ demonstrated no distinct change in the NH_3_ yield rate (Figure 9E,F).

It has recently been reported in the literature that noble metal nanocrystals (NCs) enclosed by high‐index facets show higher NRR performance owing to the strong adsorption of the NRR intermediate to the surface site located at a step than at a flat terrace.^[^ ^177^, ^178^, ^183^ ^]^ Jiang et al. fabricated high‐index faceted Au great stellated dodecahedra (GSD) using a double‐step potential method in a choline chloride‐urea (ChCl‐U)‐based aging deep eutectic solvent (DES).^[^ ^177^ ^]^ TEM analysis confirmed that the high‐index (331) planes were dominant in the GSD Au NCs. Using the catalyst, the highest average NH_3_ yield rate (49.9 μg h^−1^ cm^−2^) and FE (28.6%) were obtained at −0.4 V_RHE_, which was superior to those of the Au spheres. In addition, 90% of both NH_3_ yield and FE were all retained during the NRR test of 6 recycling at −0.4 V_RHE_. DFT calculations were conducted for the entire catalytic cycle of NRR on the three Au surfaces. In all Au surfaces, the first proton–electron transfer to N_2_ yielding N_2_H^*^ (N_2_ + ^*^ + H^+^ + e^–^ → N_2_H^*^) was RDS and NRR activity in the order of Au(110) > Au(331) > Au(111). They explained that the (110) step of the (331) facets is a highly active site for the NRR. Noble metal catalysts with high‐index facets for the NRR have also been studied for Pt‐based catalysts.^[^ ^178^ ^]^ Mao et al. reported excavated cubic Pt–Ir alloy NCs with high‐index (710) facets prepared by CV electrodeposition. The Pt_93_Ir_7_ alloy NCs exhibited higher NRR activity than Pt and Pt/C in terms of NH_3_ formation rate (28 μg h^−1^ cm^−2^) and FE (28.6%) at −0.3 V_RHE_ in 1 mM HCl. Furthermore, Pt_93_Ir_7_ alloy NCs presented high stability in NH_3_ production rate and the FE during 6 consecutive cycles at −0.3 V_RHE_. DFT calculations supported that the Pt‐stepped surface‐modified Ir atom suppressed the HER and facilitated the first proton–electron transfer to adsorbed N_2_ into N_2_H^*^.

### 2.6 Non‐noble‐metal‐based electrodes for NRR

**FIGURE 10:**
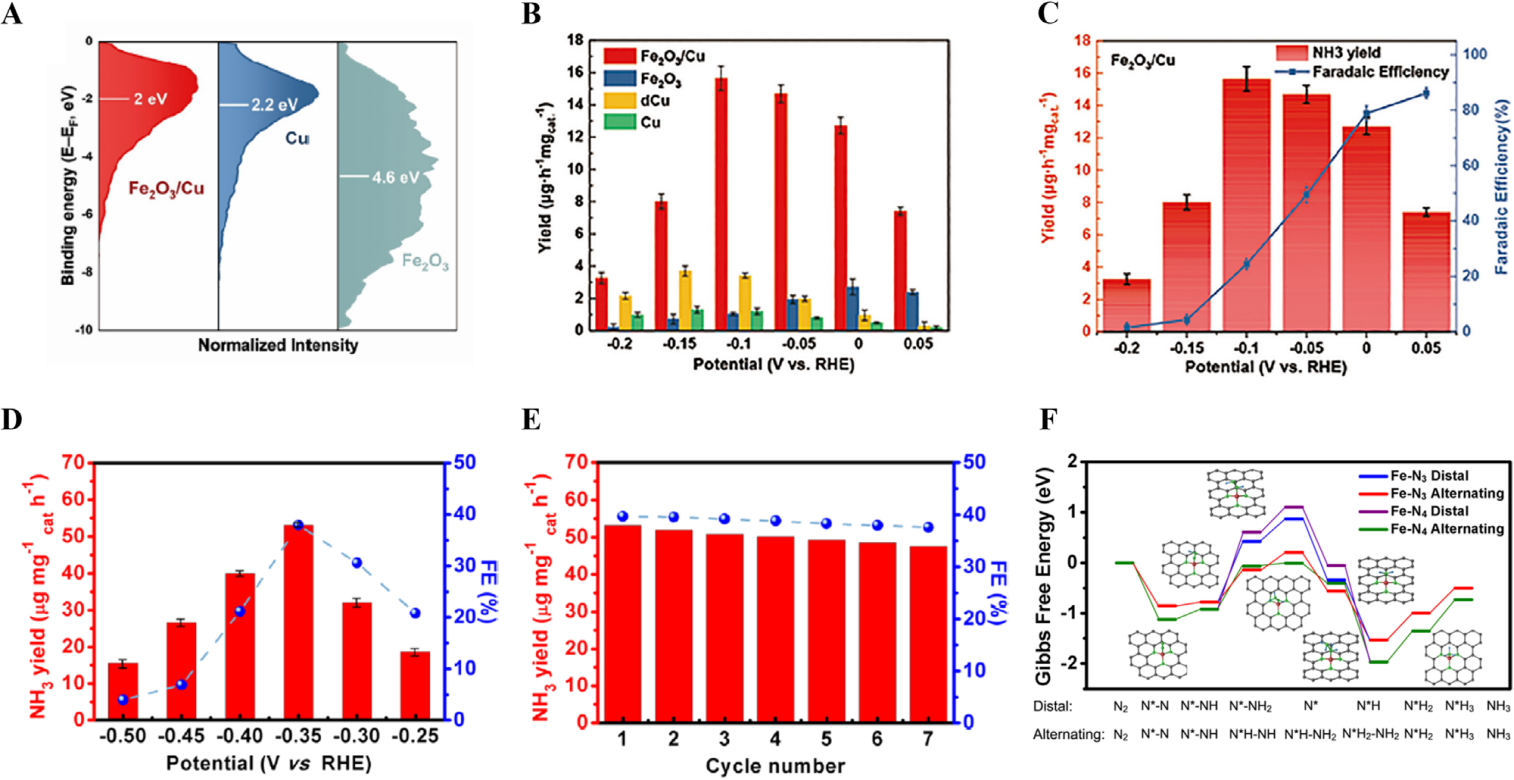
Self‐supported non noble metal‐based electrodes for NRR. (A) Valence band spectra of Fe_2_O_3_/Cu, Fe_2_O_3_, and Cu. (B) The NH_3_ yield of Fe_2_O_3_/Cu, Fe_2_O_3_, and Cu at different potential in 0.1 M KOH. (C) The NH_3_ yield rate and FEs of the Fe_2_O_3_/Cu at different potential in 0.1 M KOH. Reproduced with permission.^[^ ^180^ ^]^ Copyright 2019, Elsevier. (D) The NH_3_ yields and FEs of the Fe SAC/N−C at different potential. (E) The NH_3_ yields and FEs of the Fe SAC/N−C at −0.35 V_RHE_ during cycling tests (F) Free energy diagram and optimized structure for the NRR on Fe−N_3_ and Fe−N_4_. Reproduced with permission.^[^ ^181^ ^]^ Copyright 2020, American Chemical Society

Non‐noble‐metal‐based electrodes also exhibit catalytic activity for the NRR. The deposition conditions and NRR activities are summarized in Table 9. For NH_3_ production from the NRR, metallic Cu NPs were prepared on a CP substrate by electrodeposition by varying the deposition time.^[^ ^179^ ^]^ The Cu catalyst electrodeposited for 2 min demonstrated a nearly monolayer shape consisting of Cu NPs on the surface of C fibers, which demonstrated the highest NRR activity with an NH_3_ formation rate of 12.0 μg h^−1^ mg^−1^ and an FE of 33% at 0 V_RHE_. During the five consecutive cycles, there was no apparent decrease in the NH_3_ formation rate and FE. The NRR activity‐correlated electronic structure of Fe_2_O_3_/Cu dendrites prepared by two‐step pulsed electrodeposition was also investigated.^[^ ^180^ ^]^ Compared with the d‐band center of Cu and Fe_2_O_3_, that of Fe_2_O_3_/Cu approached the Fermi level, indicating the strong chemisorption of the reaction intermediate (Figure 10A). As a result, Fe_2_O_3_/Cu exhibited the highest NRR performance in all potential ranges compared to commercial Cu, Fe_2_O_3_, and electrodeposited Cu (Figure 10B). The highest NH_3_ yield rate of Fe_2_O_3_/Cu was 15.66 μg h^−1^ mg_cat_^−1^ at −0.1 V_RHE_, with an FE of 24.4% (Figure 10C). The enhanced NRR activity of Fe_2_O_3_/Cu was not ascribed to the enhancement in conductivity or charge‐transfer properties but to the increased catalytically active sites and nitrogen adsorption. P‐block elements can be employed as NRR catalysts.^[^ ^182^ ^]^ Sun et al. prepared Bi_2_O_3_ nanoplates on the surface of functionalized exfoliated graphene (FEG) by an electrochemical method. The FEG was fabricated by electrochemical exfoliation of graphite foil. The Bi_2_O_3_ nanoplates were then electrodeposited onto the FEG. The Bi_2_O_3_/FEG electrode exhibited a high NH_3_ yield rate of 5.68 μg h^−1^ mg_Bi_^−1^ with an FE of 11.2% in 0.1 M Na_2_SO_4_ at −0.5 V_RHE_. They explained that the high NRR activity of Bi_2_O_3_/FEG was attributed to the Bi center from Bi_2_O_3_, which provided strong binding of ^*^N_2_H as well as facile electron and mass transfers. Single‐atom catalysts (SACs) have demonstrated their potential as efficient catalysts for the NRR.^[^ ^181^ ^]^ Yang et al. fabricated Fe single atoms anchored on nitrogen‐doped carbon (Fe SAC/N−C) by electrodeposition and subsequent annealing in a N_2_ atmosphere. The Fe atoms were atomically dispersed on the N−C substrate, as confirmed by the extended X‐ray absorption fine structure. The highest NH_3_ yield rate of 53.1 μg h^−1^ mg_cat_^−1^ was obtained with an FE of 39.6% at −0.35 V_RHE_ (Figure 10D). In addition, consecutive cycling tests exhibited the high stability of Fe SAC/N–C (Figure 10E). It was revealed that the NRR on Fe SAC/N−C followed an alternating reaction pathway, whereas the Fe−N_3_ coordination on Fe SACs could suppress the competing HER and decrease the reaction energy barrier during the NRR, as supported by DFT calculations (Figure 10F).

## 3 CONCLUSION AND OUTLOOK

Recently, the development of electrocatalysts for energy conversion systems has attracted significant attention because they can mitigate environmental pollution and produce valuable energy resources such as hydrogen, hydrocarbon compounds, and ammonia. Among electrocatalysts, self‐supported electrodes have advantages for application in energy conversion systems, such as minimizing the ohmic resistance owing to the binder‐free structure, fast electron transfer from seamless contact between the electrocatalyst and substrate interface, high catalyst utilization, and avoidance of catalyst agglomeration during the electrochemical reaction. Electrodeposition is one of the representative methods for self‐supported electrode fabrication that can be conducted in a one‐pot system under mild conditions. Furthermore, it is easy to modulate the compositional and morphological properties of the electrocatalyst by controlling the deposition parameters, such as electrolyte concentration, deposition potential/current, and deposition time. To this end, many studies on self‐supported electrodes fabricated by electrodeposition have been conducted. Herein, we summarize the electrocatalysts prepared by electrodeposition for the electrochemical reactions of various energy conversion systems.

The electrodeposition method, which can easily control the composition and morphology of an electrocatalyst, shows the strength of the electrochemical reaction. Notably, the reaction intermediates play a crucial role in the electrochemical reaction, and their binding energies depend on the surface properties of the electrocatalyst. In the case of the HER, a suitable H binding energy on the catalyst surface directly provides the high intrinsic activity of the electrocatalyst. During the ECR, the final product is determined according to the binding strength of the CO_2_^*–^ intermediate on the catalyst surface. In the electrochemical NRR, because the N_2_ intermediate is strongly adsorbed on the catalyst surface, a high catalytic performance can be achieved. Therefore, realizing specific surface properties by electrodeposition leads to enhanced catalytic performance. Furthermore, increasing the ECSA of the electrocatalyst by a simple electrodeposition method is feasible, which can provide a number of active sites for electrochemical reactions. Improving the active sites can overcome the drawbacks of the inferior intrinsic activity of transition‐metal‐based catalysts. The synergistic effect between the specific composition and the enlarged ECSA of the electrocatalyst improves the catalytic performance. A stability test of the electrocatalyst should also be considered to determine the catalytic performance. A powdered catalyst anchored onto a substrate by a polymer binder suffers from the catalyst peeling problem during the electrochemical reaction, which leads to degradation of the catalyst performance. In catalysts prepared by electrodeposition, the binder‐free electrode configuration provides a strong connection between the catalyst and substrate, resulting in stable performance under vigorous operating conditions.

Although the electrocatalysts prepared by electrodeposition exhibit reasonable catalytic performance, there are remaining challenges to be considered. First, the electrodeposition is only applicable on conductive substrate, thus it is necessary to develop highly conductive and corrosion‐resistant substrate. The other concerning point is that it is difficult to achieve elaborate control of nano‐sized structure, crystallinity, and stoichiometry of materials using electrodeposition method alone. In this sense, recent studies on electrodeposited catalyst have reported the effect of post‐treatment methods such as electrochemical etching, sulfidation, phosphidation, and annealing on the electrodeposits. Furthermore, investigation on advanced electrodeposition technology combining with other synthesis method is required to overcome the limitations of traditional electrodeposition method (e.g., hydrothermal‐^[^ ^184^, ^185^ ^]^ or microwave‐assisted^[^ ^186^, ^187^ ^]^ electrodeposition). In addition, the operando/in‐situ characterization during the electrochemical reaction is beneficial to understand the behavior of reaction species, which can provide the direction of optimizing the electrocatalyst development. Through the operando/in‐situ characterization, monitoring the morphological and compositional changes is possible in real time, which can observe the proper electronic structure affecting catalytic activity.

Meanwhile, it is also important to develop an electrode for the commercialization of energy conversion systems. As a first step, understanding the electrode structure is essential for the operation of practical energy conversion systems such as water electrolyzers and fuel cells. Since the GDE facilitates diffusion of the reactants to the electrocatalyst surface and the emission of products, optimizing the porosity, wettability, and gas permeability of GDE is required to enhance the mass‐transfer behavior in energy conversion systems, especially in high‐current‐density region. Moreover, self‐supported electrodes with porous substrates such as CP, Ti paper, and Ni foam can be directly employed as GDE and using such a substrate with high electric conductivity reduces the electrolyzer system resistance. Second, large‐scale electrodes are required to maximize system performance and enhance efficiency. The electrodeposition method for self‐supported electrode fabrication makes it easy to scale‐up the electrode area, and it can be directly applied as the GDE for commercially available energy conversion systems. Improving the stability of electrocatalysts is also a significant challenge for commercialization. Commercial energy conversion systems are operated in the high current range under strong acidic and alkaline conditions, which can mechanically or chemically damage the electrocatalysts and degrade the system performance. Therefore, it is necessary to improve the catalyst corrosion resistance as well as establish suitable operating conditions for energy conversion systems. In summary, recent studies demonstrate that the electrodeposition is an efficient method to fabricate self‐supported electrode under mild condition. To be a promising electrode for commercially available energy conversion system, further efforts on the elaborate control of electrodeposits, the optimization of electrode properties (e.g., porosity, wettability, gas permeability, etc.), scale‐up of electrode area, and improvement of long‐term stability still remain as challenges.

## CONFLICT OF INTEREST

The authors declare no conflict of interest.
